# Abundance or stress? Faunal exploitation patterns and subsistence strategies: The case study of Brush Hut 1 at Ohalo II, a submerged 23,000-year-old camp in the Sea of Galilee, Israel

**DOI:** 10.1371/journal.pone.0262434

**Published:** 2022-01-26

**Authors:** Tikvah Steiner, Rebecca Biton, Dani Nadel, Florent Rivals, Rivka Rabinovich

**Affiliations:** 1 Institute of Archaeology, The Hebrew University of Jerusalem, Jerusalem, Israel; 2 Department of Bible, Archaeology and the Ancient Near East, Ben-Gurion University of The Negev, Beer-Sheva, Israel; 3 Zinman Institute of Archaeology, University of Haifa, Mount Carmel, Haifa, Israel; 4 ICREA, Barcelona, Spain; 5 Institut Català de Paleoecologia Humana i Evolució Social (IPHES), Tarragona, Spain; 6 Departament d’Història i Història de l’Art, Universitat Rovira i Virgili, Tarragona, Spain; 7 Institute of Earth Sciences, National Natural History Collections, The Hebrew University of Jerusalem, Jerusalem, Israel; University at Buffalo - The State University of New York, UNITED STATES

## Abstract

The submerged site of Ohalo II was occupied during the Last Glacial Maximum (LGM), between 23,500–22,500 cal BP, bridging the Upper Paleolithic/Epipaleolithic transition in the southern Levant. The site is known for the excellent preservation of its brush huts and botanical remains. This study examines the behavior of its past inhabitants through analysis of the entire faunal assemblage found on the three successive floors of Brush Hut 1. Furthermore, it provides an opportunity to test differing models of prey choice and assess whether the observed resource diversification is the result of resource depression (explained by Optimal Foraging Theory) or resource abundance (explained by Niche Construction Theory). We focused on a quantitative, qualitative and spatial investigation of the more than 20,000 faunal remains, combining traditional zooarchaeological methods with microwear analysis of teeth and Fourier Transform Infrared Spectroscopy (FTIR) of burnt bones. Identification of faunal remains to the most detailed level possible, combined with analysis of skeletal element frequencies allowed reconstruction of a profile of the desired prey, highlighting the importance of small, expedient prey compared to larger game (ungulates). FTIR was used to identify degrees of burning and to develop a key to identifying burnt bones from water-logged environments. Availability of multiple food sources within a rich habitat may have driven exploitation of those varied local resources, rather than targeting energetically-rich large prey. The choice of a littoral habitat that could be intensively exploited is an example of niche selection. Comparison with contemporaneous and later sites contributes to the ongoing discussion about Early Epipaleolithic prey choice, and the impact, if any, of the LGM in the Jordan Valley. Ohalo II is an example of diverse prey choice motivated by abundance rather than stress, at a 23,000-year-old fisher-hunter-gatherers camp.

## Introduction

The final Pleistocene zooarchaeological record in the Mediterranean southern Levant is generally characterized by a shift from hunting of large animals (“higher-ranked”) to hunting smaller, more difficult to attain prey (“lower-ranked”) [1–6]. The number of fallow deer, a common prey species, decreased in favor of smaller ungulates (predominantly gazelle) and a variety of small prey. This is particularly evident through the Epipalaeolithic period in the Mediterranean Levant, where the Early and Middle Epipaleolithic (ca. 24,500–15,000 cal BP) encompass the Kebaran and Geometric Kebaran cultures, respectively, while the Late Epipalaeolithic encompasses the Natufian culture [1, 5–7]. Population growth and over-exploitation of resources in the Levant is argued to have driven foragers to broaden their dietary range and procure a wider variety of plant and animal resources, including small carnivores, birds, fish, mollusks and wild cereals and legumes. The appearance and proliferation of ground stone tools for plant processing point to the growing importance of plant food during the Upper Paleolithic and especially during the Epipaleolithic [5, 8–11].

The diversification and intensification in the subsistence base of Late Pleistocene hunter-gatherers was coined the Broad Spectrum Revolution (BSR) [12, 13]. An increase in dietary breadth is explained by two opposing approaches, one suggesting it was the outcome of resource depletion, the other suggesting the impact of resource abundance. Optimal Foraging Theory (OFT), a concept from the world of behavioral ecology, assumes that foragers pursue prey based on net energy return after cost of capture and processing. New resources are added to the diet only when there is a decline in preferred, high-caloric return options. Researchers further defined prey categories based not only on biological systematics, but rather on their physical and behavioral characteristics, dividing small prey animals into slow game (e.g. tortoises) and quick game (e.g., birds and hares), whereby foragers select prey on the basis of cost of capture vs. net caloric return [1, 2, 14, 15]. The shift between acquisition of slow small prey and quick small prey is interpreted as an indicator of resource diversification due to resource depression and demographic growth during the Epipaleolithic [1, 2, 4–6].

Optimal Foraging Theory (OFT) is a framework whereby hunter-gatherers were theoretically forced to adapt to resource depression and the declining availability of preferred food species by broadening their resource base [16]. When human population surpassed a certain threshold, hunting pressure on energetically-rich prey types would increase, causing their decline and thus lowering their availability and forcing exploitation of more varied prey [3, 5]. However, some sites have a broad range of nutritional resources and exhibit little to no signs of resource depletion [16].

Niche Construction Theory (NCT) posits that resource intensification results from active engagement with the environment, rather than mainly reaction to external conditions [16, 17]. Within this theory, populations are drawn to zones of resource abundance and stability, where they utilize a wide range (or a broad spectrum) of available resources that could support the population over time. Multiple food options may also encourage experimentation with new plant and animal resources. One driving force behind resource diversification is risk avoidance, i.e., improving predictability and reducing uncertainty. An environment of abundant resources and little demographic and climatic pressure can drive resource diversification and encourage prolonged occupation or repeated visits to a particular site. The two theories are not mutually exclusive; long term depletion of high-ranking prey due to population growth or climatic change may occur in some sites, while optimally-located sites continue to exploit abundant resources.

The excellent preservation of faunal and botanical remains at the water-logged site of Ohalo II provides a unique opportunity for a detailed analysis of subsistence strategies during the Last Glacial Maximum (LGM), ca. 23,000 cal BP, a period encompassing the shift from the Upper Paleolithic to the Epipaleolithic. Furthermore, the well-preserved and rich faunal assemblage from Brush Hut 1 offers an opportunity to explore which of the above scenarios best explains the structure of the faunal assemblage. The expected faunal signatures for each model are laid out in Table 1. Under OFT, we would expect to see signs of resource depression and diversification, while under NCT we could expect to see resource abundance and diversification. The signatures may overlap or be interpreted in different ways in some cases; therefore, a cautionary approach is adopted, with the understanding that an assemblage may not align with one single model, especially in the case of a single site, as presented here. However, some of the criteria are more indicative of a NCT-influenced assemblage, such as the presence of both high-ranking large ungulates and high-ranking small game, and the concurrent exploitation of diverse sources rather than dependence on a narrow range of species (gazelle or fallow deer).

**Table 1:** Expected faunal signatures for Optimal Foraging Theory and Niche Construction Theory.

Optimal Foraging Theory—Signs of resource depression [2–5, 14, 15, 18]	Niche Construction Theory—Signs of resource abundance [16, 17, 19, 20]
Dominance of small game vs. large game; Shift from small, slow game in favour of small, quick game	Presence of both high-ranking large ungulates and high-ranking small, slow game
Dominance of gazelle vs. fallow deer	Diverse array of food resources exploited concurrently: large and small mammals, fish, birds and plants
Intensive processing of adult gazelle bones	A range of processing techniques may be seen
Presence of very young juvenile gazelles	Presence of mainly prime-aged ungulates
Decrease in size and number of tortoises	Tortoises remain a steady resource with size unrelated to over-predation
Shift to include lower-ranked marine resources	Exploitation of diverse marine resources as part of varied diet
Increased use of plant resources	Increased use of plant resources
Increased mobility of human populations	Decreased mobility of human populations

### Ohalo II

**Fig 1:**
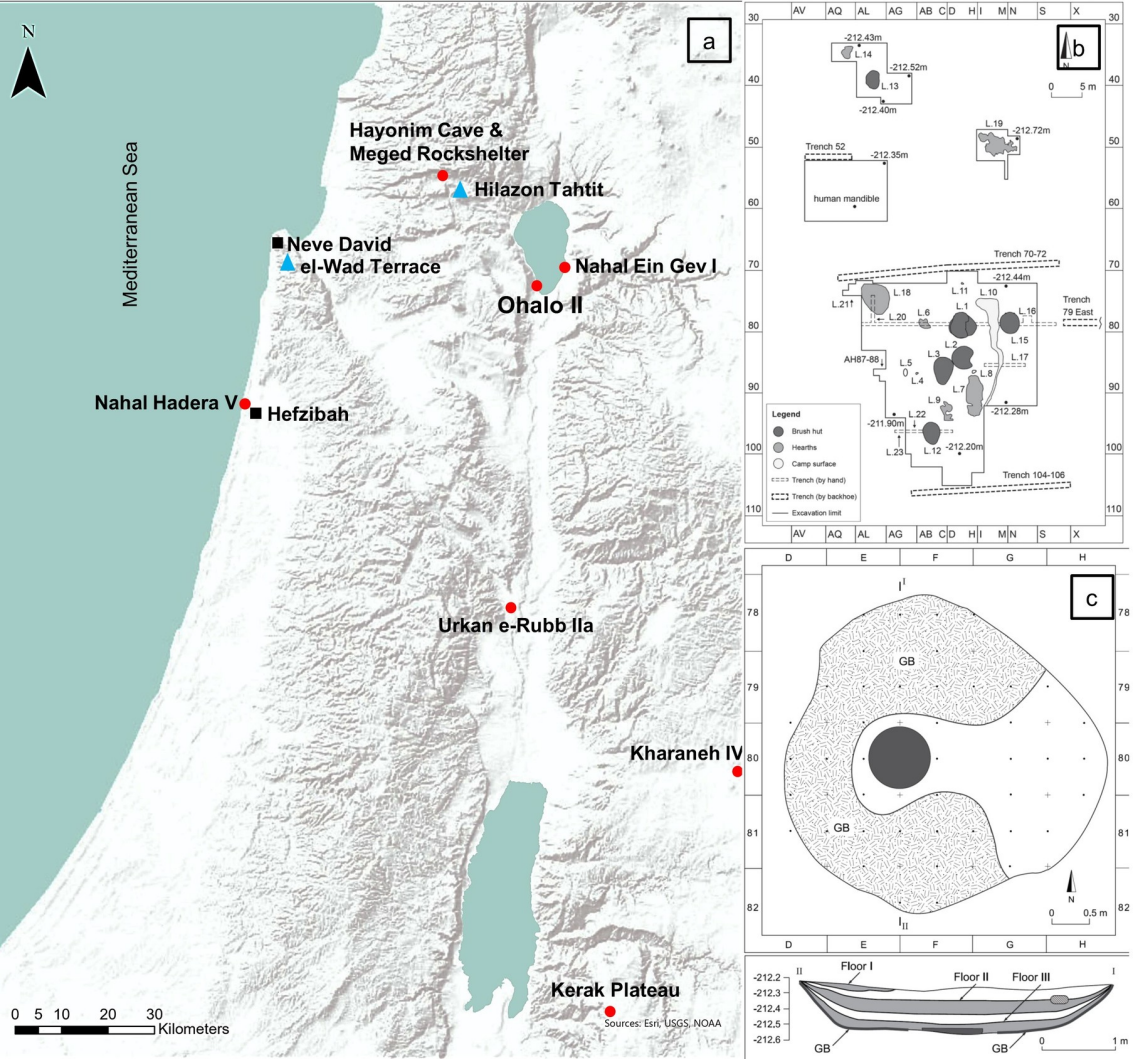
Map with location of southern Levantine Epipaleolithic sites mentioned in the text (a), plan of Ohalo II (b) and plan of Brush Hut 1 (c). Early Epipaleolithic sites are marked with red circles, Middle Epipaleolithic with black squares and Late Epipaleolithic with blue triangles.

The site of Ohalo II was discovered in 1989, following drought conditions that lowered the water level of the Sea of Galilee by several meters. Excavations were carried out between 1989–1991, and again between 1998–2001. The site is located near the southern tip of the modern Sea of Galilee, about 9 km south of Tiberias (Fig 1A and 1B). The quick inundation of the site and the ensuing deposition of fine silt led to outstanding preservation of the submerged camp [21–23]. The site covers ca. 2000 m^2^ and includes the remains of six oval-shaped brush huts, open-air hearths, a grave of an adult male [24], various installations and middens. Abundant organic and inorganic materials provide a wealth of information about the lifeways of fisher-hunter-gatherers during the LGM [10, 22, 25–30]. Over 40 ^14^C dates sampled from 14 features range between 22,500–23,500 cal B.P. [31, 32].

Brush Hut 1 is the largest of the six brush huts found at the site, measuring 4.5 × 3.0 m, with a long north-south axis and entrance from the east. Three successive floors were excavated, with the top surface layer (Floor I) only partially preserved due to erosion and disturbance. Floor II (middle layer; ca. 12 m^2^) and Floor III (bottom layer; ca. 14 m^2^) were completely excavated (Fig 1C). The floors were separated by irregular layers of silt and sand, 3–5 cm thick on average, indicating short accumulation periods between them, most likely no more than several seasons, if at all [22]. The hut is ca. 30 cm deep at its center [23]. Within the hut, ca. 60,000 charred plant remains were studied from Floor II [10, 25], and ca. 55,000 from Floor III [29]. Many flint tools and debitage [32, 33], bone tools [34], and numerous faunal remains of mammal [28, 35], birds [36] and fish [30, 37] were also recovered. Floor III was covered, except for a hearth in the middle, with grass bedding laid in an overlapping pattern over ca. 7 m^2^ of the floor. A clay substance may have been used as an adhesive material for the grass, creating a mat [22].

Large quantities of wild cereal grains were identified within the hut. Wild emmer (*Triticum dicoccoides)* and wild barley (*Hordeum spontaneum)*, along with oats (*Avena sterilis*) and other cereals, were gathered in the vicinity of the site and made up an important part of the Ohalo II diet [38]. Analysis of starch grains preserved on a grinding stone on Floor II indicates that it was used to grind larger-grained barley, wheat, and oats [27, 39]. Spatial analysis of cereal grains on Floors II and III identified clusters of grains and activity areas [10, 29]. The cereal harvesting method is evident from microwear analysis of composite sickle blades [40].

Previous work identified over 12,000 fish bones in Brush Hut 1, with 94% belonging to the Cyprinidae (minnow and carp) and 6% to the Cichlidae (tilapia) families [30, 37, 41]. The presence of all anatomical regions, the skeletal part frequencies and the species richness and diversity all indicate an artisanal fishing camp engaged in intensive aquatic activities [30]. The remains of twisted cords [42] and dozens of notched stones interpreted as weights for fishing nets [43] suggest that fishing was practiced at the camp. However, it is important to consider the influence of natural fish death assemblages in this water-logged site [44]. Floors I and II of Brush Hut 1 show an over-representation of *Mirogrex terraesanctae*, a small pelagic fish regarded as bland and of low economic value; its presence may be due to natural death assemblages and not to cultural preference [30].

The rich bird assemblage reflects various environments, including fresh water, field, scrub-forest, cliff and Mediterranean [45]. The presence of terminal phalanges and wing elements suggest collection of wings for ritualistic or symbolic purpose [36].

As Ohalo II was submerged for millennia and the preservation of features (especially brush hut floors) with their in situ contents is well-documented, the site served and still serves as a case-study for a range of research questions pertaining to camp life during the LGM. In this paper, the main objectives include identification of faunal remains from an entire well-preserved brush hut (Brush Hut 1) to the most detailed possible level; mesowear and microwear analysis of ungulate teeth; and identification of spatial patterning of the faunal remains across the three floors. Additionally, we address the methodological issue of identifying burnt bones in a water-logged site using Fourier Transform Infrared Spectroscopy (FTIR) to assess degrees of burning and build a framework to classify burnt bones on the basis of surface color. This multidisciplinary study provides an opportunity to evaluate the OFT and NCT theories of resource diversification within the context of a complete and well-preserved faunal assemblage.

## Methods

Brush Hut 1 was excavated in 0.5 × 0.5 × 0.05 m units. All excavated material was wet-sieved through a 2 mm mesh during the first two seasons, and a 1 mm mesh in the subsequent seasons. The faunal assemblage is stored in the National Natural History Collections of the Hebrew University, Jerusalem (NNHC-HUJ), and was studied using the NNHC-HUJ comparative collection. The bones from each floor were studied as a discrete assemblage, enabling characterization of each assemblage and comparison between them. When possible, mammal and reptile bones were identified to species level; otherwise, they were assigned to body-size groups [46] (Table 2). The avifauna was identified to family level only. The basic unit for quantifying bones from which other calculations are derived is NISP (Number of Identified Specimens).

**Table 2:** Body Size Group (BSG) classification used in this study.

Body Size Groups (example species)	Body mass (kg)
BSGA *(Bos primigenius)*	>1000
BSGB (*Dama mesopotamica*)	80–250
BSGC (*Sus scrofa)*	40–80
BSGD (*Gazella gazella)*	15–40
BSGE (*Vulpes vulpes*)	1–15

Minimum Number of Individuals (MNI) and Minimum Number of Elements (MNE) counts were calculated for each floor as a separate unit [47]. Small game/ungulate ratios measure dietary breadth, by dividing the number (NISP) of small game by the sum of small game and ungulate remains [2]. Small game refers to BSGE mammals, birds, and Testudines. Richness and evenness values of the studied taxa were calculated using the Shannon Weaver Diversity Index [48, 49] and Simpson’s Reciprocal index [14, 50, 51].

Minimum Animal Units (MAU) of gazelle/BSGD and fallow deer/BSGB were plotted against the Modified General Food Utility Index (MGUI) values for sheep and caribou respectively [52, 53]. MAU were derived from dividing the MNE by the number of times the element appears in the skeleton of a given species. Bone mineral density of pronghorn antelope and reindeer [53] were correlated with %survivorship for fallow deer and gazelle. The NISP/MNE ratio is a proxy for measuring intensity of fragmentation [47]. All bone surfaces were examined under a microscope (1–40×) to identify any taphonomic or anthropogenic modifications, and the location and quantity of each was recorded. Anthropogenic marks due to exploitation and processing include cut marks [54–57], percussion marks [58–60], long bone shaft breakage [61, 62] and split phalanges [18, 63].

Gazelle aging is based on mandibular wear stages recorded for tooth sequences [46, 64] and individual teeth [64]. Fallow deer mandibular wear stages were recorded for tooth sequences [65]. A general aging scheme according to Stiner [66] was used to place individual ungulate teeth or sequences into juvenile, adult, and old adult categories.

Epiphyseal fusion was examined in order to determine age [64, 67]. Anatomical measurements [68, 69] were plotted to determine sex ratios for gazelles and their relative size in comparison with other Levantine sites. In the absence of sex-diagnostic skeletal elements, gazelle bone measurements were used to identify sex using a discriminant function analysis developed by Munro et al. [70]. Size of tortoises was measured by the mean humeral breadth, taken from the medio-lateral breadth of the humerus shaft at its narrowest point.

Tooth mesowear is a proxy for the dietary signal averaged over an animal’s lifetime [71]. Dental microwear indicates the diet during the last days of life of an individual [72]. The combination of these two proxies provides insights into two different time scales of an animal’s life, allowing for inference of a potential seasonal signal in the assemblage. To reduce inter-observer error, dental mesowear and microwear analyses were conducted by a single experienced researcher (FR).

We used the standardized mesowear analysis method introduced by Mihlbachler et al. [73], based on seven cusp categories (numbered from 0 to 6), ranging in shape from high and sharp (stage 0) to completely blunt with no relief (stage 6). The average value of the mesowear data from a single sample of fossil dentitions corresponds to the ‘mesowear score’ or MWS (ibid.). Unworn (and marginally worn) teeth, extremely worn teeth, and those with broken or damaged cusp apices were omitted from mesowear analysis [74].

Tooth microwear was analysed following the protocol established by Solounias and Semprebon [75]. The tooth crowns were cleaned with acetone, then ethanol 96%, to remove any residue of preparatory adhesives from the surface and to eliminate any remaining sediments or dust. The surfaces were molded using high-resolution dental silicone (Heraeus Kulzer, PROVIL novo Vinylpolysiloxane, Light C.D. 2 regular set) and casts were created using clear epoxy resin (C.T.S. Spain, EPO 150 + K151). All molded teeth were carefully screened under a stereomicroscope, discarding those presenting evidence of taphonomic alteration [76, 77]. Casts were observed with a Zeiss Stemi 2000C stereomicroscope at 35× magnification, using the refractive properties of the transparent cast to reveal microfeatures on the enamel. Microwear scars were quantified in two areas—on the paracone of the upper molars and the protoconid of the lower molars—using an ocular reticule of 0.16 mm^2^. We used the classification of features defined by Solounias and Semprebon [75] and Semprebon et al. [78] which distinguishes pits and scratches. The presence of cross scratches, hyper-coarse scratches, gouges, large pits and puncture pits was recorded qualitatively (presence/absence). In addition, scratch textures were assessed using the scratch width score (SWS) which is obtained by giving a score of ’0’ to a tooth with predominantly fine scratches, ’1’ to a tooth with a mixture of fine and coarse types of textures, and ’2’ to a tooth with predominantly coarse scratches. Individual scores for a sample of teeth were then averaged to get the SWS.

The application of FTIR analysis on burnt or diagenetic bones is common in the Paleolithic Levantine archaeological record [15, 79–84]. Burn codes based on bone surface color [15, 85, 86] may not apply to a water-logged site, where bones are often dark colored and stained due to their unique depositional environment. Thus, the Ohalo II faunal assemblage presented an opportunity to test the use of FTIR to identify burning and exposure to intense heat in a water-logged archaeological site. Samples of brown, dark brown, black, grey, white, and orange bone were selected from Floors III and II of Brush Hut 1, five bones of each color from each floor. Colors were determined with the Munsell soil color chart [87], as follows: brown (strong brown/brown, 7.5YR 4/2, 4, 6), dark brown (dark brown, 7.5YR 3/2, 4), orange (reddish yellow, 7.5 YR 6/6, 8), black (black, 2, 3/0), grey (light grey/grey, 7.5 YR 4, 5, 6, 7/0), and white (7.5YR, 8/0). The sample of bones was ground with potassium bromide (KBr) then pressed into pellet using a Pike Technologies hand press. The infrared spectra were obtained using a Thermo-scientific Nicolet iS5 Spectrometer, controlled by OMNIC software. All equipment was thoroughly cleaned between samples to prevent cross-contamination and kept under heat lamps to prevent moisture. The FTIR analysis was done in the Department of Structural Biology at the Weizmann Institute, Israel.

All specimens were recorded in the database within their 0.5 × 0.5 m excavation unit (subsquare). Using ArcGIS 10, the specimens were then mapped on their floor and presented on a grid of 0.25 m^2^ units. The templates used are similar to the plant distribution maps of Brush Hut 1 from Weiss et al. [38] and Snir et al. [29], for purposes of comparison. The sum of all specimens in each subsquare was then divided by the volume of excavated sediment in the same subsquare to account for variation in excavation depth and provide accurate densities.

## Results

### Taxonomic composition and diversity

About 20,000 bones from Brush Hut 1 were examined. A total of 4406 specimens from all three floors were identified to species and body group level, resulting in a 22% identification rate (Table 3). Gazelle and gazelle-sized mammals (BSGD) (NISP = 1056; 25.3%) were the most common mammalian taxa. The second largest group was fallow deer and similar size mammals (BSGB) (NISP = 524; 12.6%). Three unidentified bone fragments belonged to a large mammal, possibly aurochs. Bones of wild boar and roe deer were also identified (NISP = 26; 0.6%). The small mammal category made up a significant portion of the faunal assemblage: fox (NISP = 228; 5.5%), hare (NISP = 80; 1.9%) and BSGE animals (NISP = 163; 3.9%) were well-represented. Small numbers (NISP = 26) of wild canid, feline and hedgehog represented 0.5% of the assemblage. Testudines (tortoises and freshwater turtles) accounted for nearly half of all identified specimens (NISP = 1729; 41.4%). Birds accounted for an additional 5.6% (NISP = 235) (Table 3).

**Table 3:** Identified faunal taxa, Brush Hut 1 (NISP).

		Floor I		Floor II		Floor III		Total	
Group	Taxon	NISP	%NISP	NISP	%NISP	NISP	%NISP	NISP	%NISP
**Ungulate**	*Gazella gazella*	68	17.4	118	10.5	329	11.4	**515**	11.7
	*Dama mesopotamica*	18	4.6	52	4.6	103	3.6	**173**	3.9
	*Sus scrofa*	1	0.3	4	0.4	13	0.5	**18**	0.4
	*Capreolus capreolus*	-	-	4	0.4	4	0.1	**8**	0.2
*Sub-total*		*87*	*22*.*3*	*178*	*15*.*9*	*449*	*15*.*6*	*714*	*16*.*2*
**Small Mammals**	*Vulpes vulpes*	10	2.6	24	2.1	194	6.7	**228**	5.2
	*Lepus capensis*	3	0.8	10	0.9	67	2.3	**80**	1.8
	*Canis* sp.	-	-	1	0.1	-	-	**1**	<
	*Felis* sp.	1	0.3	2	0.2	1	<	**4**	0.1
	*Erinaceus* sp.	2	0.5	5	0.4	9	0.3	**16**	0.4
*Sub-total*		*16*	*4*.*2*	*42*	*3*.*7*	*271*	*9*.*3*	*101*	*7*.*5*
**Body Size Groups**	BSGA	1	0.3	-	-	2	0.1	**3**	0.1
	BSGB	23	5.9	106	9.4	222	7.7	**351**	8.0
	BSGD	93	23.8	243	21.5	547	18.9	**883**	20.0
	BSGE	18	4.6	24	2.1	121	4.2	**163**	3.7
*Sub-total*		*135*	*34*.*6*	*373*	*33*	*892*	*30*.*9*	*1400*	*31*.*1*
**Testudines**	*Testudo graeca*	59	15.1	304	26.9	492	17.0	**855**	19.4
	*Mauremys* sp.	2	0.5	3	0.3	11	0.4	**16**	0.4
	Testudines	75	19.2	194	17.2	589	20.4	**858**	19.5
*Sub-total*		*136*	*34*.*8*	*501*	*44*.*4*	*1092*	*37*.*8*	*1729*	*39*.*3*
**Birds**	Podicepididae	2	0.5	1	0.1	15	0.5	**18**	0.4
	Anatidae	-	-	2	0.2	16	0.6	**18**	0.4
	Laridae	1	0.3	-	-	1	<	**2**	0.1
	Rallidae	1	0.3	-	-	2	0.1	**3**	0.1
	Falconidae	1	0.3	3	0.3	3	0.1	**7**	0.2
	Accipitridae	2	0.5	3	0.3	8	0.3	**13**	0.3
	Phasianide	2	0.5	7	0.6	11	0.4	**20**	0.5
	Corvidae	1	0.3	5	0.4	18	0.6	**24**	0.5
	Passeriformes	-	-	6	0.5	36	1.2	**42**	1.0
	Und. Birds	6	1.5	8	0.7	73	2.6	**87**	2.0
*Sub-total*		*16*	*4*.*2*	*35*	*3*.*1*	*182*	*6*.*4*	*234*	*5*.*5*
**Total**		**390**	100.0	**1129**	100.0	**2888**	100.0	**4406**	100.0

Und. = unidentified to specific species or family; < = less than 0.1%.

**Fig 2:**
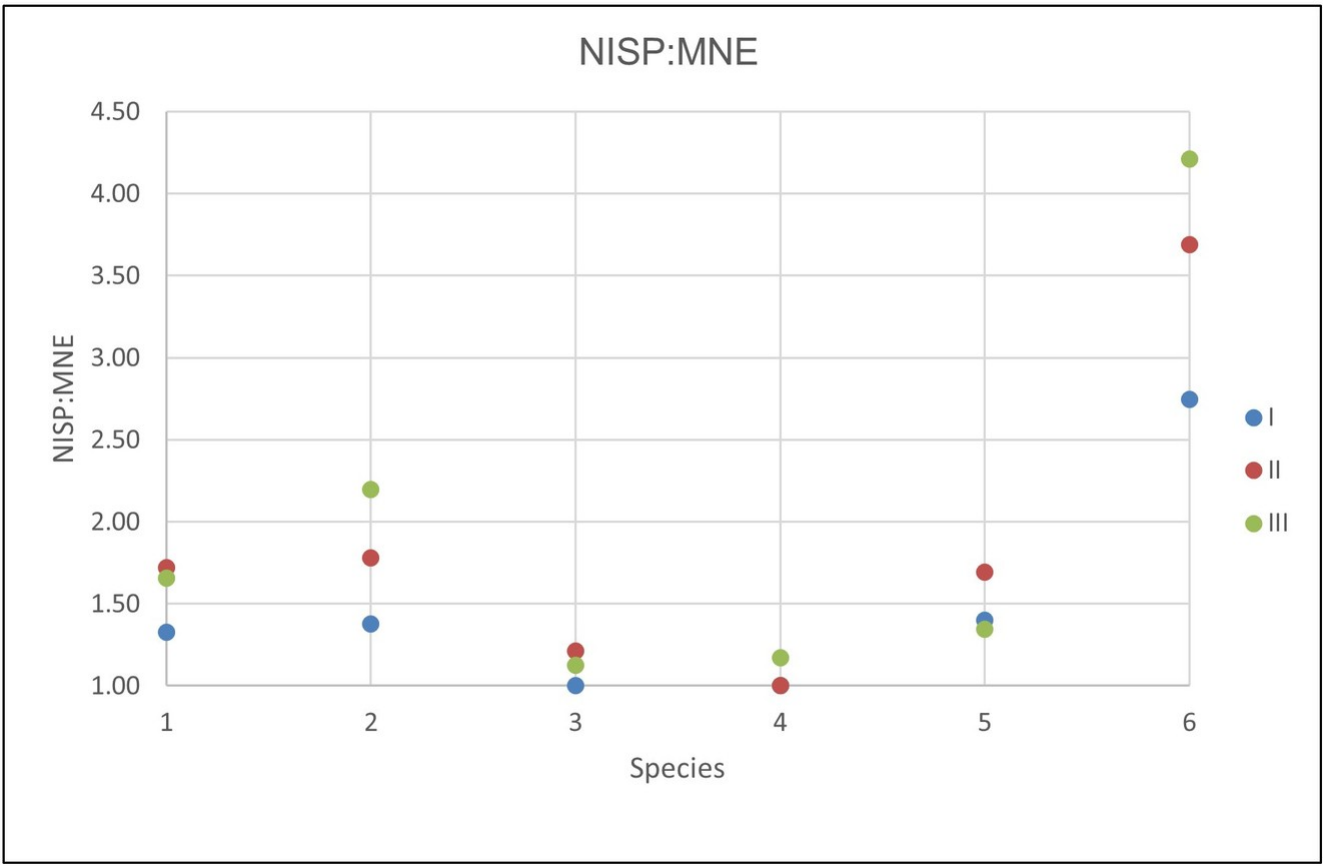
NISP/MNE ratios from Brush Hut 1, by floor. 1 = Gazelle/BSGD, 2 = Fallow Deer/BSGB, 3 = Fox, 4 = Hare, 5 = BSGE, 6 = Tortoise. The higher the ratio, the more fragmented the assemblage.

Floor I, the uppermost layer, was disturbed, partially eroded, and only yielded 9% of the identified specimens. Floor II covers 12 m^2^ and contained 26% of the identified specimens. The remaining 65% were found on Floor III, which measures 14 m^2^. The species representation was similar on all three floors, although the MNI and MNE were highest on Floor III for nearly all taxa (Table 4). The NISP/MNE ratio varied between the floors (Fig 2). Floor III showed the highest rate of bone fragmentation, especially in the gazelle/BSGD group.

**Table 4:** MNI and MNE counts of identified taxa (excluding birds) from Brush Hut 1.

Species	Floor I		Floor II		Floor III		Total	
	MNI	MNE	MNI	MNE	MNI	MNE	MNI	MNE
*Gazella gazella*	5	54	6	84	9	259	20	397
*BSGD*	-	28	-	52	-	131	-	211
*Dama mesopotamica*	4	16	3	39	5	53	12	108
*BSGB*	-	13	-	34	-	65	-	112
*Sus scrofa*	1	1	1	4	1	13	3	18
*Capreolus capreolus*	-	-	1	4	1	4	2	8
*Vulpes vulpes*	1	12	1	19	4	149	6	180
*Lepus capensis*	1	2	1	9	2	47	4	58
*Canis* sp.	-	-	1	1	-	-	1	1
*Felis* sp.	1	1	1	2	1	1	2	4
*Erinaceus* sp.	1	2	1	5	1	9	3	16
*BSGE*	-	10	-	13	-	58	-	81
*Testudo graeca*	2	32	4	116	9	222	15	370
*Mauremys* sp.	1	2	1	3	1	11	3	16
*Testudines*	-	15	-	19	-	33	-	67
**Total**	**17**		**21**		**33**		**71**	

The small game/ungulate index showed differing proportions between the floors (Table 5). The numbers of small game (NISP; small mammals, BSGE, Testudines and birds) rise from Floor II (NISP = 602, 53%) to Floor III (NISP = 1667, 57%), with Testudines increasing to 38% of the total assemblage on Floor III. The small game component was more significant on Floor III and decreased in the subsequent layers, while accordingly the medium and large mammal components increased. Ungulates accounted for 42% of the Floor III assemblage, and 52% of Floor I.

**Table 5:** Small game/ungulate index from Brush Hut 1.

Floor	Ungulates NISP	Small Game NISP	Testudines NISP	Total	Small Game/ Ungulate Index
Floor I	52% (204)	13% (50)	35% (136)	**390**	0.47
Floor II	47% (527)	9% (101)	44% (501)	**1129**	0.53
Floor III	42% (1220)	20% (575)	38% (1092)	**2887**	0.57

Small game/ungulate ratios measure dietary breadth, by dividing the number (NISP) of small game by the sum of small game and ungulate remains [2]. Testudines are shown separately to emphasize their dominance in this category but calculated as small game in the index. Small game includes small mammals (Table 1), BSGE and birds.

However, when comparing the NISP of the floors, the proportions are not drastically different. Gazelle and BSGD were 30% of Floor III and 32% of Floor II. Fallow deer and BSGB were 11% of Floor III and 14% of Floor II. There was a small increase through time in both gazelle and fallow deer procurement, and a decrease in small, quick game from Floor III to II; the numbers of tortoise specimens remain relatively steady over time.

Calculations of richness and evenness values using the Shannon Weaver Diversity Index and Simpson’s Reciprocal index showed that the Brush Hut 1 assemblage was diverse (Table 6). Equitability (V) values indicate a relatively even distribution of taxa. Floor III was still relatively diverse, but less so than the other floors because of the high numbers of gazelle and tortoise (*T*. *graeca*), the two most abundant species.

**Table 6:** Diversity indices from Brush Hut 1, by floors.

Floor	Shannon Weaver Diversity Index (H)	Equitability (V)	Simpson’s Reciprocal Index (D)
I	1.947	0.78	9.5
II	2.011	0.81	8.7
III	1.915	0.77	6.6

Higher H value means a more diverse and equally distributed assemblage. Equitability values close to 1.0 indicate more even distribution of taxa. Simpson’s Reciprocal Index starts from 1, representing an assemblage or community with only one species. The higher the value, the greater the diversity. The numbers from Brush Hut 1 are calculated using MNI.

The total NISP of gazelle in the Brush Hut 1 assemblage is 515 (11.7%) and BSGD is 883 (20%), forming the most dominant mammal group with a combined NISP of 1398 (31.7%) (Table 2). Minimum numbers of elements (MNE) and minimum animal units (MAU) were calculated for all anatomical elements of gazelle and BSGD (S1 and S2 Tables in S1 File for skeletal element abundance).

**Fig 3:**
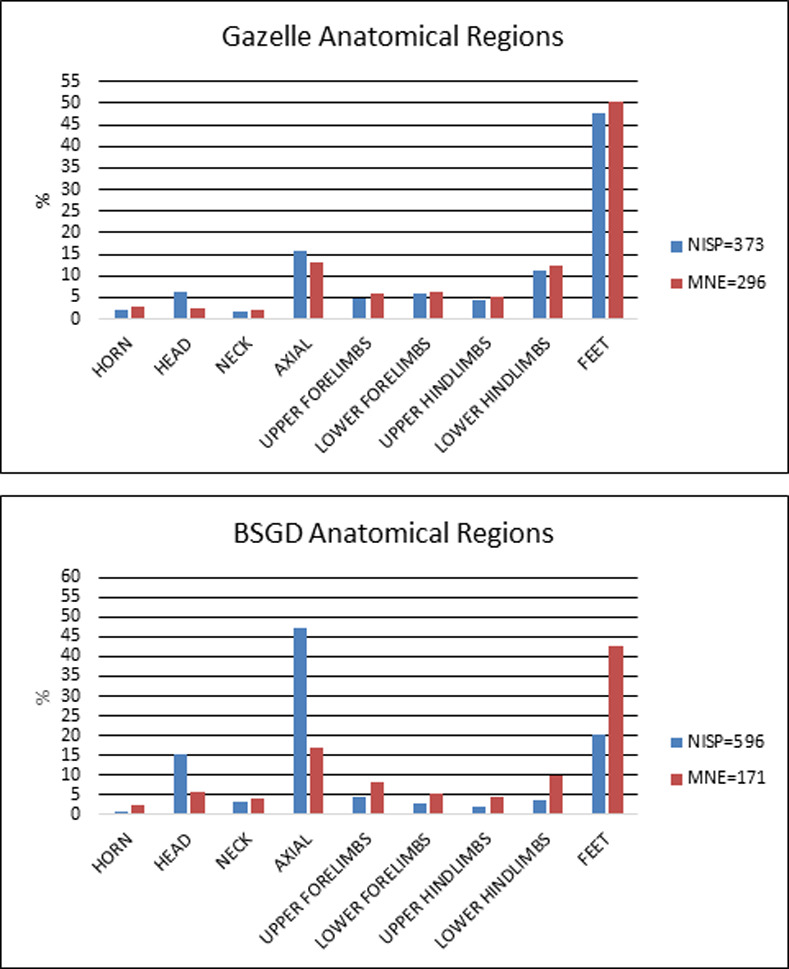
Identified skeletal elements of gazelle and BSGD by anatomical region in Brush Hut 1.

The most common gazelle skeletal elements in the Brush Hut 1 assemblage were feet, which include carpals, tarsals, and phalanges. In the BSGD group axial fragments are the most common, encompassing ribs and vertebrae (Fig 3). Distal humeri were the most abundant long bone element, followed by distal tibia and proximal femur, all of which are considered meat-bearing joints while the feet bones are not [52]. The low numbers of axial elements and skulls identified as gazelle results from identification bias whereby skull, vertebrae and rib fragments were difficult to assign to species level, and therefore were classified by body-size only. However, they attest to the presence of the entire skeleton in the brush hut.

**Fig 4:**
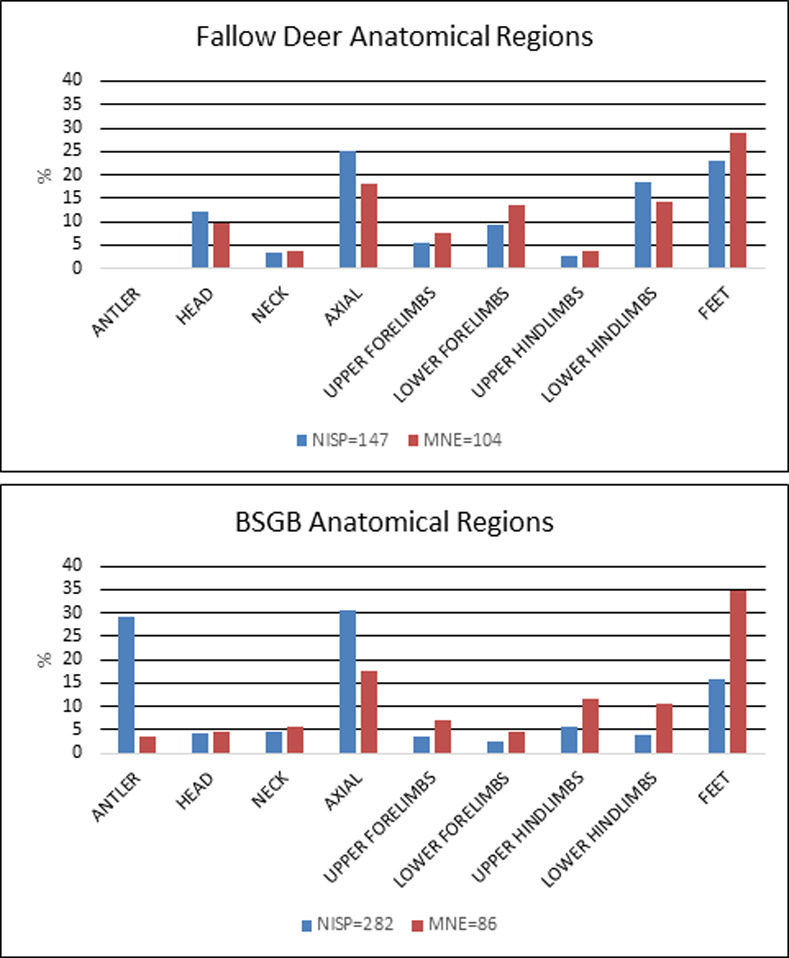
Identified skeletal elements of fallow deer and BSGB by anatomical region in Brush Hut 1.

Fallow deer (*Dama mesopotamica*) and BSGB ungulates (80–250 kg), which were likely the largest fauna hunted at Ohalo II, represent 11.9% (NISP = 524) of the assemblage. The most common elements were lower hindlimbs and feet, followed by axial elements and antler fragments (Fig 4 and S4 and S5 Tables in S1 File). In general, more fallow deer long bone elements and cranial elements were present than those of gazelle. Fragmented axial elements were 28.7% of the total NISP, followed by antlers (average fragment size: 22 × 6 mm) that constituted 19.1% of the NISP. The ability to easily identify antler fragments accounts for the overabundance of head elements. The entire skeleton is represented in the assemblage.

The most common small mammal is the fox (*Vulpes vulpes*), constituting 5% of the entire faunal assemblage, and 6.7% of the assemblage on Floor III. Foxes were more common on Floor III (NISP = 104; MNI = 4) than on Floor II (NISP = 24; MNI = 1). Elements from most anatomical regions were found in Brush Hut 1, indicating the presence of whole animals in the hut, with feet as the most common element (S6 Table in S1 File). An entire articulated paw was found on Floor III. Hare (*Lepus capensis*) constitutes 1.8% (NISP = 80) of the brush hut assemblage. Feet accounted for 54% of the identified skeletal elements. Again, most of the hare remains were on Floor III (NISP = 67).

The previously studied portion of the Brush Hut 1 bird assemblage includes seasonally-migrating birds and reflects multiple environments: fresh water, field, scrub-forest, cliff, and Mediterranean shore [36, 45]. An additional 234 bird elements from Brush Hut 1 were identified in this study (Table 3). The largest identified group was Passeriformes (songbirds), followed by waterfowl (Podicepididae, Anatidae, Laridae, Rallidae), birds of prey (Falconidae, Accipitridae), and ground-feeding birds (Phasianidae).

Testudines are the most abundant identified taxa in the brush hut (NISP = 1729; 39.3% of the total assemblage), probably due to the easily fragmented nature of the carapace and the ability to easily identify even the smallest shell fragments. The two identified species are *Testudo graeca* (Spur-thighed Tortoise) and *Mauremys* cf. *rivulata* (Western Caspian Turtle). *T*. *graeca* constitutes 19.4% (NISP = 855) of the faunal assemblage, *M*. cf. *rivulata*, 0.4% (NISP = 16), and unidentified Testudines 19.5% (NISP = 858). The overwhelming majority of unidentified Testudines are likely Spur-thighed Tortoise whose shell fragments are too small to assign to a specific species. The assemblage consists of 87% carapace, 9% plastron, and 4% long bones. In order to assess the size of the tortoises exploited at Ohalo II we compared the size of the *T*. *graeca* humeri to *T*. *graeca* specimens from the osteological comparative collections at the NNHC-HUJ. We measured 11 modern tortoise specimens from the NNHC-HUJ, for which full carapace length, width and humeral breadth were available (S7 Table in S1 File). Carapace length and width correlate strongly with humeral breadth, and therefore we could estimate the approximate size of the Ohalo II tortoises based on humeral size. The mean breadth of 19 humeri from Brush Hut 1 is 2.9 mm (SD = 0.5), with a maximum of 3.57 mm and a minimum of 1.82 mm. According to our comparison between the modern specimens and the ones from Brush Hut 1, the tortoises measured between 90–125 mm long and 70–95 mm wide. The largest tortoise humerus in Brush Hut 1 (mean breadth 3.57 mm) probably originated from an individual not exceeding 140 mm in carapace length. The maximum length of extant *T*. *graeca* shells in northern Israel reaches 213–258 mm [88]. The Ohalo II Testudine assemblage is remarkably uniform in size, characterized by the small size of the tortoises. An additional species of tortoise, *Testudo kleinmanni*, is found in the Negev desert of southern Israel [88], and is characterized by a smaller average shell length of 130 mm. We ruled it out on the basis of morphological distinction from the Ohalo II tortoises, which lack the notched nuchal element with a W-shaped anterior edge that characterizes *T*. *kleinmanni* [89].

### Skeletal element processing

We examined the body parts distribution by species and body size groups using various indexes. Gazelle and BSGD ungulates (%MAU) were plotted against Binford’s [52] normed utility indices for sheep [data from 47]. The correlation between gazelle %MAU and a “high” utility index is negative and insignificant (Spearman’s rho = -0.21; R² = 0.0057, p = 0.19), and the same is true for BSGD ungulates (Spearman’s rho = -0.06; R² = 0.068, p = 0.71). The results showed a “reverse-utility” curve, with high quantities of low utility elements present, mainly feet (S1 Fig in S1 File). Some high utility humeri and femurs were present too, but the skeletal elements clearly do not correlate with a positive utility index. It is important to note that the %MAU calculations do not include unidentified axial elements, and thus these are not represented in the utility curve. Therefore, a highly fragmented assemblage such as the Brush Hut 1 example is less suited to categorizations of utility in this manner, as a significant portion (i.e., ribs and vertebrae) could not be identified to specific elements. No statistical correlation was found between food utility and %MAU for either fallow deer or BSGB (Spearman’s rho = 0.040; p = 0.79), although high utility proximal and distal femurs were present on Floors II and III (S2 Fig in S1 File).

Gazelle bone survivorship plotted against bone density values [47, 61, 90] did not indicate density mediated attrition, with survivorship frequency lower than 50% for nearly all elements, regardless of bone densities (S3 Fig in S1 File). The correlation coefficient between the two variables was insignificant (Spearman’s rho = 0.056, p = 0.69). When tested for each floor, again there was no significant correlation between bone density and survivorship. It should be noted that the average length of identified gazelle bones, 22.5 mm, and their average width, 7.4 mm, was consistent over the floors (S4 Fig in S1 File). No correlation was found between bone mineral density and skeletal element survivorship for fallow deer either (Spearman’s rho = -0.007; p = 0.96). Patterns of exploitation and breakage seem to be quite similar for both gazelle and fallow deer. The rate of fragmentation probably affected the rate of skeletal element survivorship more than any other factor. The average length and width of identified fallow deer skeletal fragments are 33 × 11 mm, and, as with gazelle, very consistent over the floors (S5 Fig in S1 File). As with the gazelle, it appears that many parts of the fallow deer were transported to the camp and heavily exploited for meat and marrow. Less feet elements were present compared to gazelles, perhaps indicating occasional removal of these elements at the kill site. Fallow deer are more commonly represented by skull elements, mainly antler and mandibles. Mandibles contain enough marrow to be consumed, though no cut marks were found on them.

**Fig 5:**
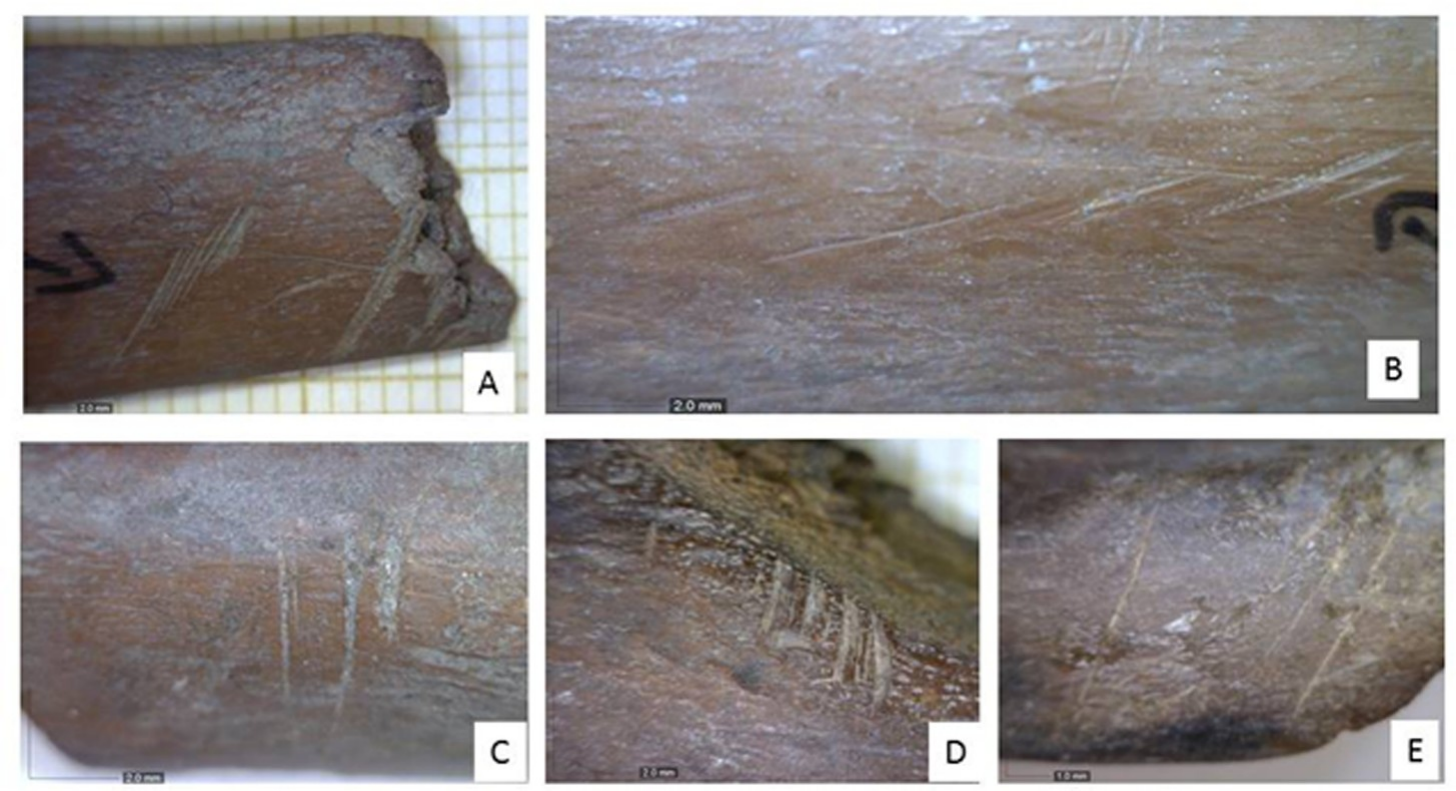
Examples of cut marks on BSGB and BSGD ungulate bones from Brush Hut 1. A: Tibia shaft, BSGB (Sp. 8176) B: Rib shaft, BSGB (Sp. 8162) C: Rib shaft, BSGD (Sp. 9402) D: Rib shaft, BSGD (Sp. 10047).

Cut marks were found on 88 specimens (2.1%), of which 87 were gazelle/BSGD (N = 57) and fallow deer/BSGB (N = 30) bones. The single outlier was on the anterior neck of a hare scapula. Cut marks were found most commonly on the ribs and long bone shafts of both species/BSG. Marks on ribs indicate disarticulation of the rib cage from the spinal column and subsequent filleting. Transverse cut marks on the proximal portion of ribs are associated with dismemberment. Skinning marks are found on the metapodials, phalanges, distal tibia and skull [56]. Dismemberment and disarticulation marks were found on joints. A cut mark on a gazelle mandible was located on the medial margin of the mandibular body, under the diastema and associated with disarticulation of the tongue. Light diagonal cuts on metapodials resulted from cleaning in preparation for marrow extraction [56] (Fig 5).

Application of direct force to the bone surface creates percussion marks, which may indicate marrow extraction [58–60]. Percussion notches, occurring most commonly on the phalanges, probably resulted from intensive marrow exploitation, which is evident from the rate of fragmentation. The first and second phalanges contain marrow that may be exploited by splitting open the bone in a longitudinal pattern [18, 63]. Phalanx splitting was identified on 33% of fallow deer/BSGB (N = 21) and 19% of gazelle/BSGD (N = 32) 1^st^ and 2^nd^ phalanges.

**Fig 6:**
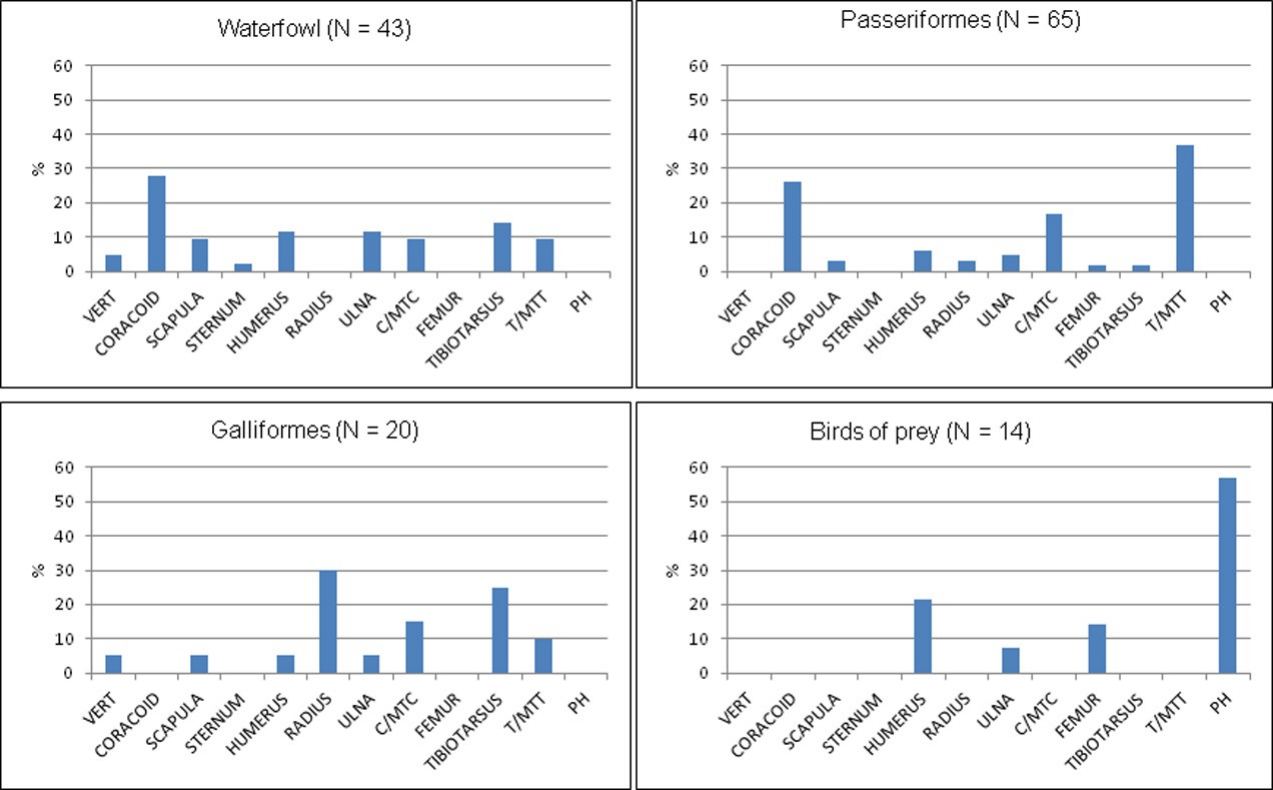
All identified elements of birds in Brush Hut 1, %NISP, by family groups. Waterfowl include Podicepididae, Anatidae, Laridae and Rallidae; Birds of prey include Falconidae and Accipitridae; ground-feeders include Phasianidae. Passeriformes are identified to order only. Vert = Vertebrae, C/MTC = Carpometacarpus, T/MTT = Tarsometatarsus, PH = phalanges.

Waterfowl were represented by almost all body parts, with many breast/wing elements which are considered meaty portions. Feet elements of birds of prey are also present (Fig 6). The species richness and skeletal element representation of birds parallel the earlier study [36].

**Fig 7:**
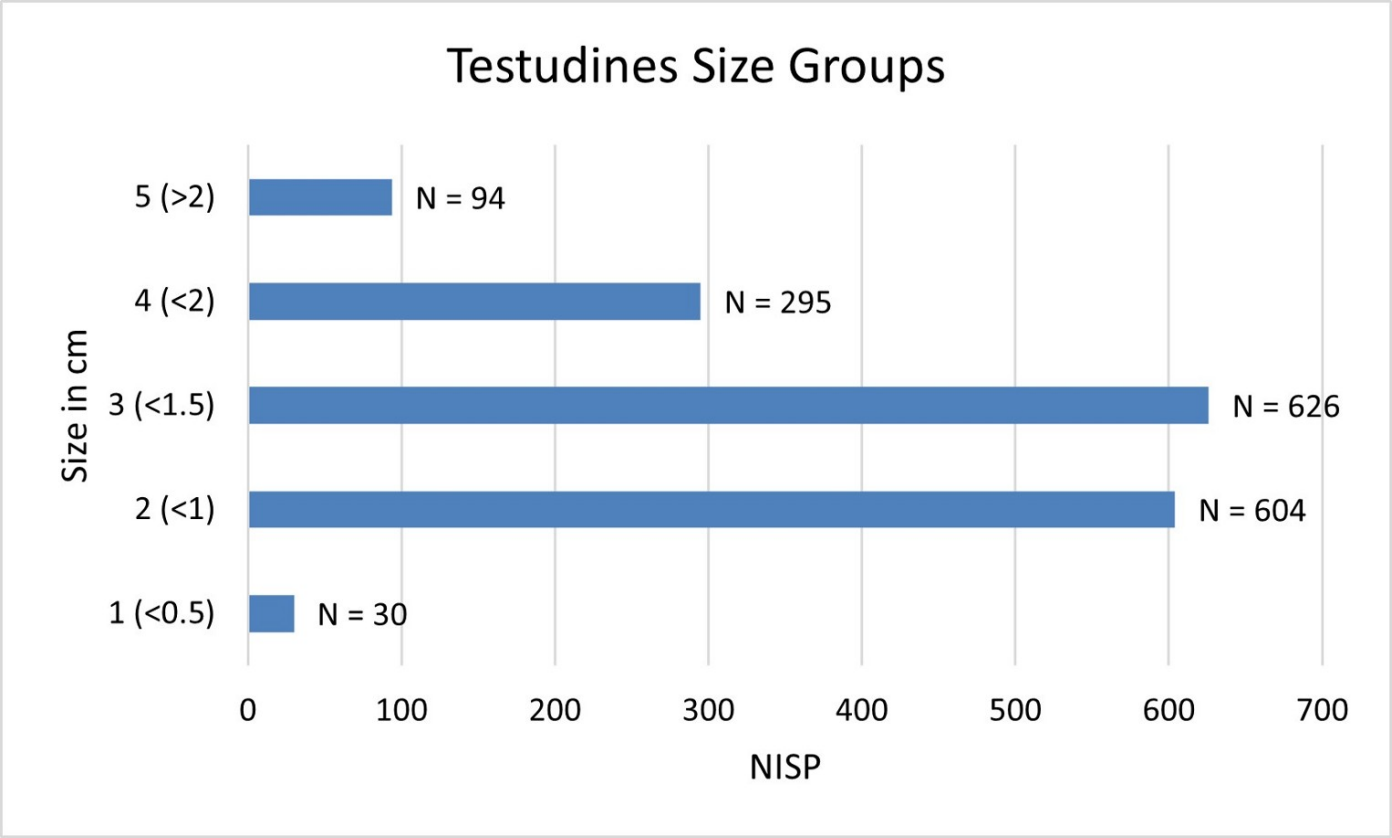
Mean size groups for tortoise plastron and carapace fragments from Brush Hut 1, all floors. Most tortoise shell pieces fall between 1.0–1.5 cm.

The Testudines assemblage is highly fragmented, with carapace the most fragmented and long bones the least. The shell fragments are small, mostly smaller than 1.5 cm, leading to the high number of fragments unidentifiable to species level (Fig 7).

Due to the small size of the identified specimens, breakage patterns and surface modification were challenging to identify. One identifiable trend, however, was the preservation of certain peripherals (bony plates running around the circumference of the carapace). Peripherals 1–3 and 7–11 were individually identified, while peripherals 4–6 were missing (S6 Fig in S1 File). High rates of impact damage on the peripherals may be attributed to a method of breaking the shell by placing it on its edge and smashing the opposite rim [15]. Bridge peripherals (peripherals 4-5-6) would be most affected by this type of impact, and those are the ones missing from the Brush Hut 1 assemblage, perhaps indicating a similar mode of exploitation. However, bridge peripherals lack thick free edges as opposed to the anterior and posterior peripherals and therefore are thinner and more fragile. As a result, bridge peripherals naturally tend to break more easily, leading to possible failure to identify them in this highly fragmented assemblage.

### Demographic profiles

Gazelle age profiles were difficult to determine from the small number of diagnostic elements, but generally prime-aged adults were exploited. Of the 80 identified gazelle teeth, 19 were whole, and only five were molars or premolars that may be given a tentative age classification. Four individual teeth belonged to gazelles under 18 months. One deciduous 3^rd^ molar was present, indicating an animal less than 15 months old. Wear stages on the available tooth sequences showed three “old adults” [66]. The teeth from Brush Hut 1 showed a pattern of few very young or very old individuals, concentrating on the prime-age prey. Floors I and II each had one juvenile gazelle (0–18 months). Floor III had at least two juvenile animals (up to 18 months), at least one very young individual (0–7 months) based on epiphyseal fusion, and at least one foetus (foetal bones NISP = 9) (S4 Table in S1 File). This variability reflects the general trend of Floor III, where more, and more varied, prey was exploited than on successive floors. There was a very small number of unfused fallow deer bones (NISP = 20; 3.8%), with 80% of them (NISP = 16) on Floor III. Based on tooth wear stages [65], most teeth represented adult animals (NISP = 24; 73%). Floors I and II had an MNI of two juveniles aged 7–14 months, and Floor III at least one juvenile specimen of 7–14 months based on tooth wear.

Limited sexual dimorphism in gazelles makes it challenging to differentiate between males and females on the basis of morphological characteristics alone [91], without specific diagnostic elements. Survivorship of diagnostic male or female elements was low in the Brush Hut 1 assemblage. Sex determinative statistical analysis [70] on a sample of 117 bones from the hut found 64 specimens to be from females (55%) and 53 from males (45%). Specifically, 69.5% of the phalanges belonged to females. The female to male ratio reflects the local herd population structure, where generally there are more adult females than males in a group [92].

### Mesowear and microwear analysis of gazelle and fallow deer teeth

Mesowear and microwear analyses were performed on dental specimens of gazelle and fallow deer from Floor III only, because of the small sample size on the other floors. The sample for mesowear consisted of 17 teeth (three fallow deer and 14 gazelle) and for microwear, 29 teeth (eight fallow deer and 13 gazelle). The mesowear scores indicate differences between the two species. Fallow deer (MWS = 2.67), with a higher mesowear score, had a more abrasive diet than gazelle (MWS = 1.36). Fallow deer is classified as a grass-dominated mixed feeder, while gazelle is a browse-dominated mixed feeder. The mesowear score of fallow deer from Ohalo II is higher than that of its modern relative from the same geographic area in Northern Israel (MWS = 1.6; [93]), while gazelles have similar values to the extant mountain gazelle (MWS = 1.2; [93]).

**Fig 8:**
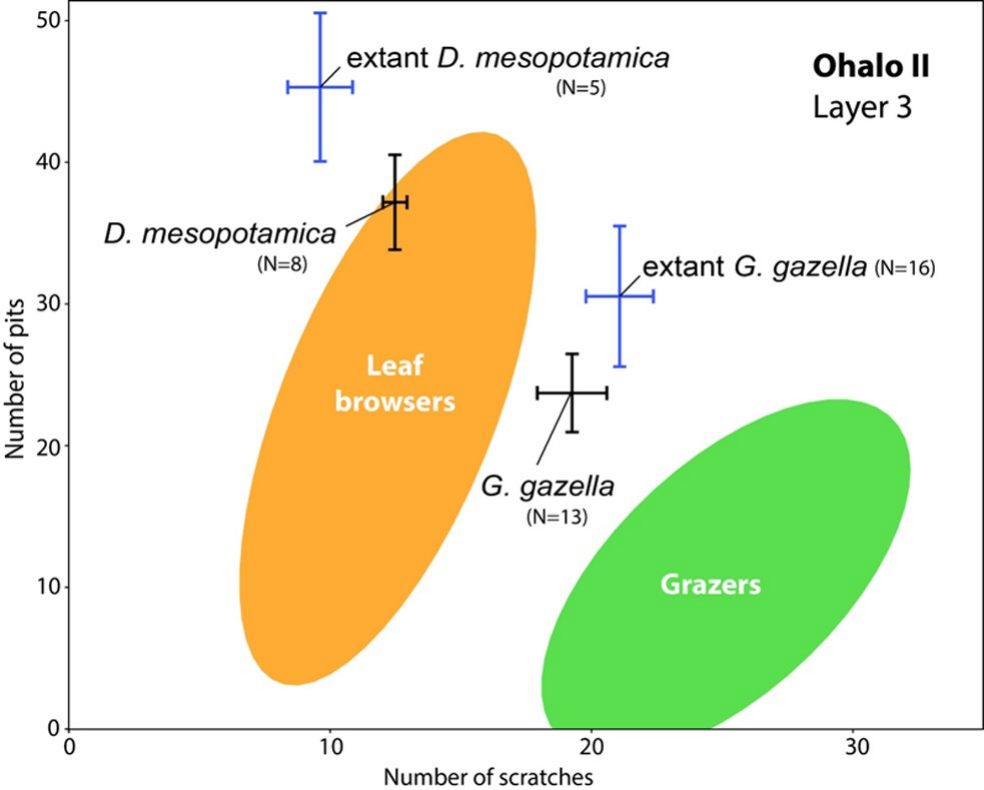
Bivariate plot of the average numbers of pits and scratches for the ungulates from floor III of Brush Hut 1 (in black) and their extant relatives (in blue; from [93]). Error bars correspond to standard error of the mean (±1 SEM) for each sample. Plain ellipses correspond to the Gaussian confidence ellipses (p = 0.95) on the centroid for the extant leaf browsers and grazers based on the reference database from [75].

The microwear patterns also show differences between the two species; however, the dietary trend reflected in this short-term signal differs from that indicated by the long-term signal of the mesowear. Fallow deer teeth have a low number of scratches that plots within the range of extant browsers. Gazelle teeth have an intermediate number of scratches that plots in between the extant browsers and grazers (Table 7 and Fig 8). Moreover, the two samples fall close to those of their modern relatives (Fig 8), suggesting similar diets at the time of death. The discrepancy between the two proxies (mesowear and microwear) for the samples from Floor III could be related to seasonal differences in diets.

**Table 7:** Summary of the mesowear and microwear data for the ungulates from floor III of Brush Hut 1.

Species		Mesowear		Microwear							
		N	MWS	N	NP	NS	%LP	%G	SWS	%XS	%HC
*Dama mesopotamica*	M	3	2.67	8	37.2	12.5	87.5	75	101	0	0
	SD		0.58		4.74	0.65					
	CV		0.22		0.13	0.05					
*Gazella gazella*	M	14	1.36	13	23.7	19.3	53.9	23.1	1.2	0	0
	SD		1.15		4.97	2.39					
	CV		0.85		0.21	0.12					

N = Number of specimens; M = Mean; SD = Standard deviation; CV = Coefficient of variation; MWS = Mesowear score; NP = Average number of pits; NS = Average number of scratches; %LP = Percentage of individuals with large pits; %G = Percentage of individuals with gouges; SWS = Scratch width score; %XS = Percentage of individuals with cross scratches; %HC = Percentage of individuals with hyper-coarse scratches. See S9 Table in S1 File for raw data.

**Fig 9:**
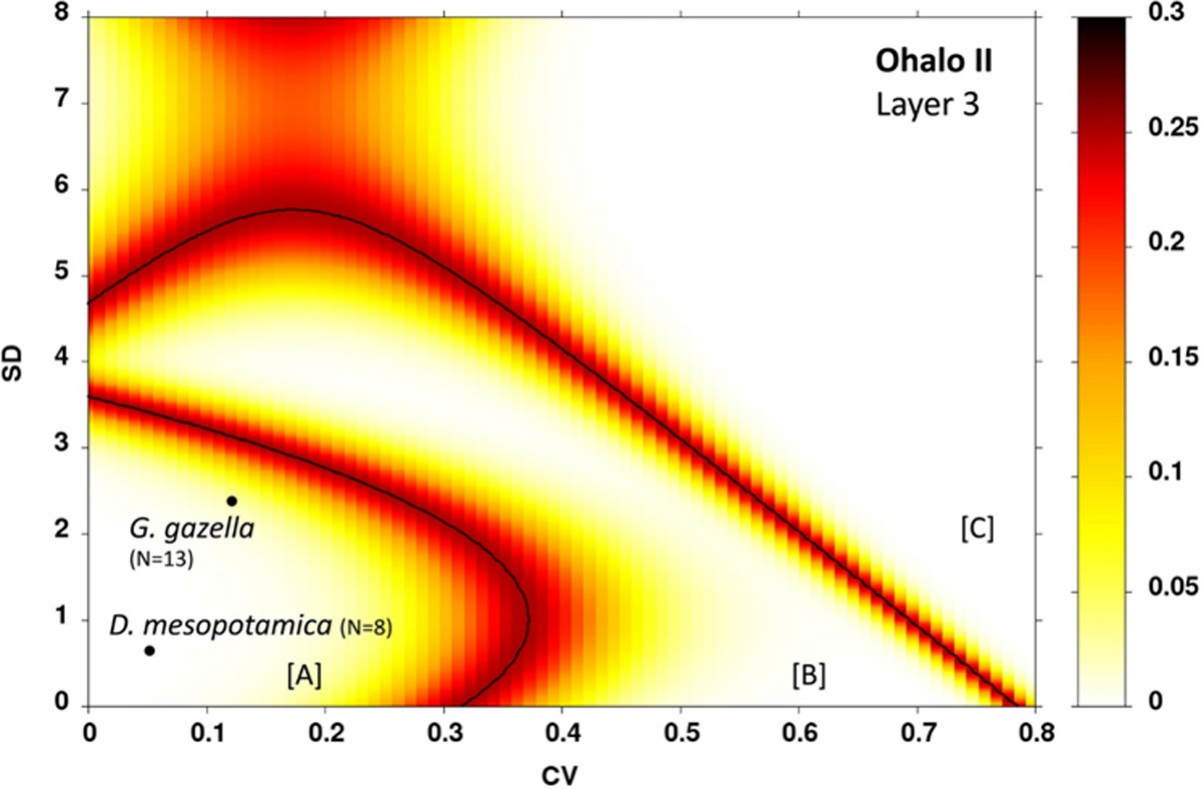
Bivariate plot of the standard deviations (SD) and coefficient of variation (CV) values of microwear data used for the classification of samples into short events (region A), long-term events (region B) or two separate short events (region C), and the boundary lines and error probability between these regions. The black dots indicate the position of the gazelle and fallow deer samples from Floor III in Brush Hut 1.

The quantification of the variability in the number of scratches shows low values for the Standard Deviation (SD) and the Coefficient of Variation (CV) for the two species (Table 7). The two samples plot in zone A of the heat map (Fig 9), i.e., in the area corresponding to a duration of accumulation shorter than three months. In comparison with modern gazelles with known date of death [93], the microwear pattern with a low number of pits and a high number of scratches fit with a death that occurred from winter to early spring.

### FTIR analysis

The FTIR spectra showed a range of bone mineral crystallization, with a rough correlation between bone surface color and splitting factor. Splitting factor (SF) is a measure of the separation of infrared peaks 604 and 565, which reflects the degree of crystallinity in carbonated apatite crystals and thus its level of preservation [94–96]. A normal range of splitting factors for modern fresh bone is 2.5–2.9, for archaeological bone 4.5–5.0 and for burnt or diagenetic bone 7.0+ [96]. The higher the SF, the larger and more ordered the apatite crystals.

**Fig 10:**
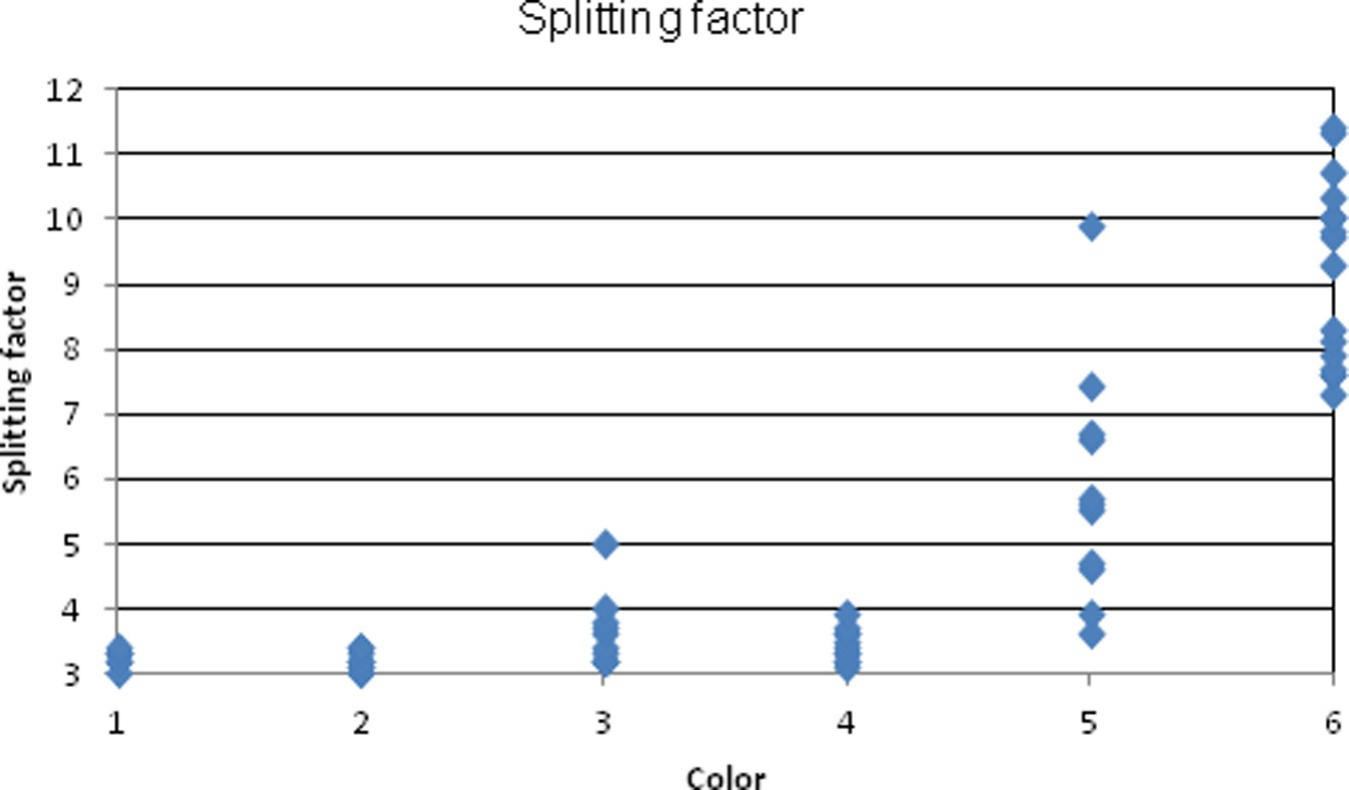
Range of splitting factors for bone samples (n = 65) from floors II and III of Brush Hut 1. Color codes: 1- Orange, 2- Brown, 3- Dark brown, 4- Black, 5- Grey, 6- White. Colors were determined with the Munsell soil color chart [87], as follows: brown (strong brown/brown, 7.5YR 4/2, 4, 6), dark brown (dark brown, 7.5YR 3/2, 4), orange (reddish yellow, 7.5 YR 6/6, 8), black (black, 2, 3/0), grey (light grey/grey, 7.5 YR 4, 5, 6, 7/0), and white (7.5YR, 8/0).

The splitting factors for the Brush Hut 1 bone samples range from 3.0 to 11.4. A SF above 8.0 is rare; the Ohalo II sample displays extremely high SF. With regards to surface color, orange, brown, dark brown and black bones all had similar SF, ranging between 3.0–5.0 (Fig 10). Infrared peaks for brown, orange, and black bones showed the presence of collagen, indicating that the bones were not exposed to high intensity fire above 700°C [97]. Black bones displayed characteristics of regular archaeological bones (3.1–3.9). The presence of carbonate peaks in brown/orange/black bones indicates a level of crystallinity reached through diagenetic factors other than heat exposure. Another indicator of bone diagenesis is the presence of fluoride [96].

**Fig 11:**
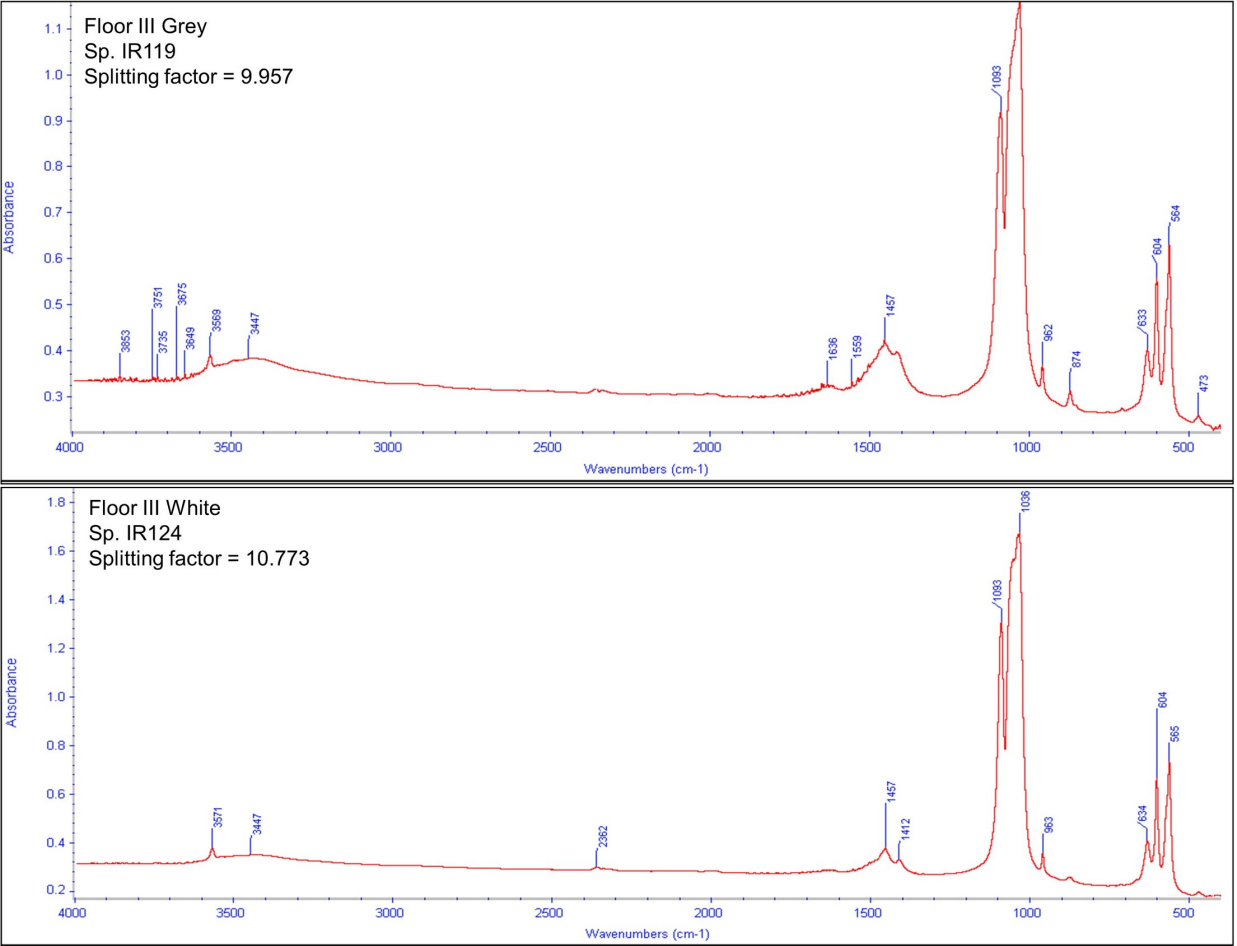
Example of infrared spectrum for grey (top) and white (bottom) bone from Brush Hut 1. The 604/565 peaks, which indicate SF and crystallinity, are clearly separated, and deeply split. The extra peak at ~633 is a reliable indicator of calcined bone [94], as is the absence of carbonate peaks at ~1458/1419. The other additional peak at ~1091 indicates highly crystalline bone apatite. The collagen peaks are absent.

Grey bones had highly variable infrared spectra, from 3.3 to 9.9. This may be due to the difficulty in identifying “grey” as this can be a combination of several surface colors. White bones were highly calcined or influenced by another diagenetic process, to a level unseen in other sites, with SF from 7.3 to 11.4. The 604/565 peaks, which indicate SF and crystallinity, are clearly separated and deeply split. The extra peak at ~633 is a reliable indicator of calcined bone [96], as is the absence of carbonate peaks at ~1458/1419. The other additional peak at ~1091 indicates highly crystalline bone apatite. The collagen peaks are absent. The infrared spectrum indicates high-intensity burning (above 650–700°C) and a resulting highly crystalline bone structure (Fig 11). Grey and white bones were intensively burnt, and other colored bones were either not burnt or only exposed to low level heat. Accordingly, ~8% (NISP = 327) of bones in Brush Hut 1 were intensively burnt. A definitive interpretation of black bones as carbonized cannot be derived from infrared spectra. Floor III was likely burnt post-occupation [26, 29]. The percentage of black bones is highest on Floor III at ~25% (NISP = 635), much higher than on other floors. Therefore, the presence of black bones may actually be because of low-intensity burning of organic materials that the hut was constructed of.

### Spatial analysis

Spatial analysis was undertaken for Floors II and III. Floor I is partially eroded and was therefore excluded. Previous spatial analyses of plant distribution on Floor II identified a cereal processing area centered around the grinding stone in the northern part of the hut, a flint-knapping area opposite the entrance [98], and an access area between the two. Floor III also showed clustering of botanical remains in the northern part of the hut, and an accumulation of “burgul” wheat around the hearth [10, 29].

**Fig 12:**
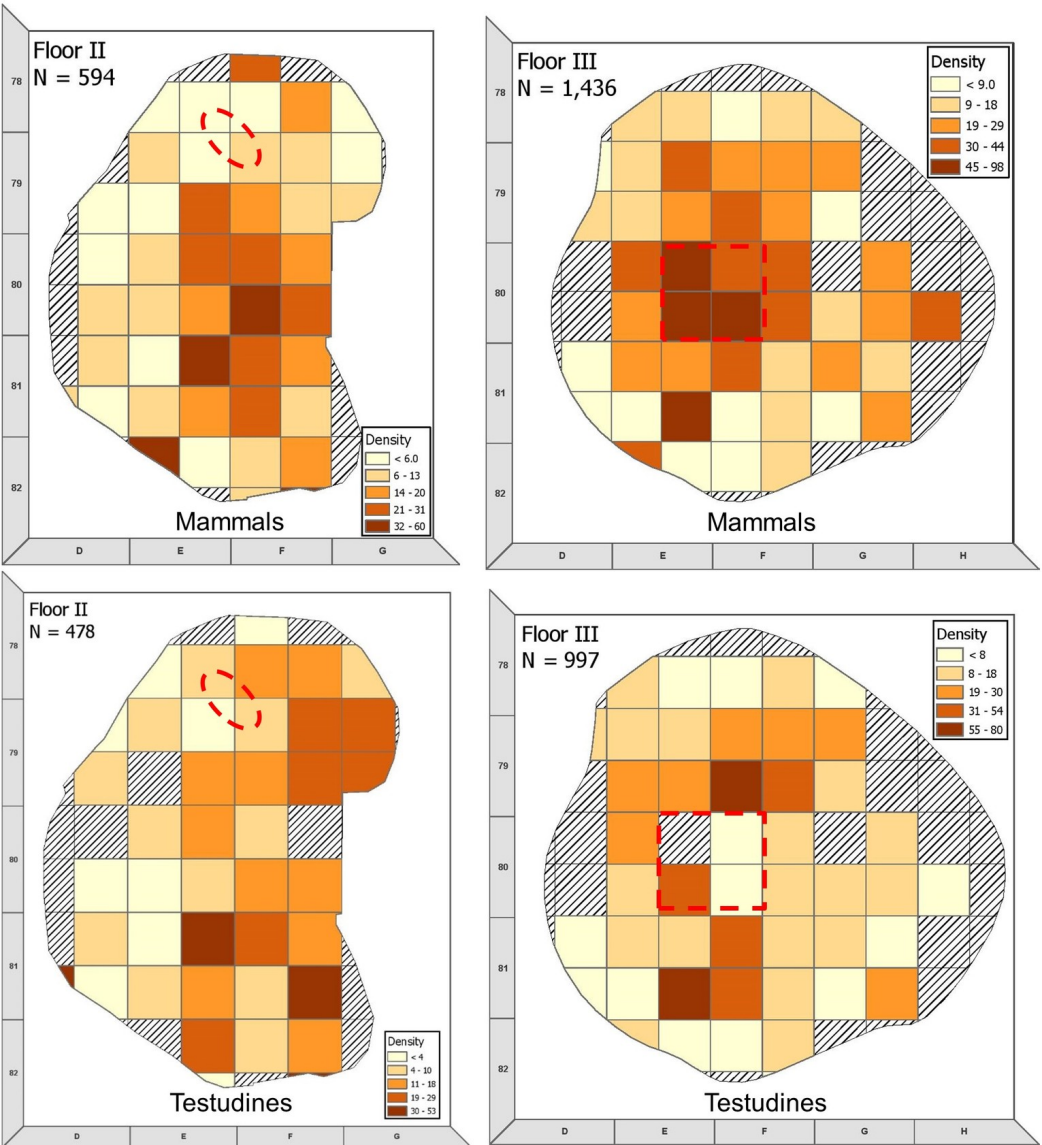
Spatial distribution of all mammalian fauna (top) and Testudines (bottom) on Floors III and II of Brush Hut 1. The sum of faunal remains per square is divided by volume of excavated sediment to resolve variation in excavation depths between squares. Hearth area on Floor III marked with red square; grinding stone on Floor II marked with red oval.

Faunal remains were distributed over most of Floor III (Fig 12), except for the edges, perhaps as a result of its bowl-like shape. Denser accumulations of bone occurred towards the center of the hut, especially in and around the hearth. The Testudines bones were distributed more evenly over Floor III, with a smaller concentration next to the hearth. There is considerable overlap in the distribution of the fauna and the botanical remains, especially the lack of botanical and faunal specimens in the eastern part of the hut and around the edges [29]. However, the bones are clustered more clearly in the vicinity of the hearth than the botanical remains. The center of the hut may have been utilized for meat preparation and/or eating, with the hearth area used for discard. This would explain the higher number of bones found there. On Floor II the mammalian fauna clusters in the center and in the southern part of the hut, but the Testudines assemblage does not seem to exhibit any spatial pattern. A distinct cluster of cereal grains around the grinding stone in the northern part of the hut suggests the separation of activity zones within the hut.

## Discussion

Previous Ohalo II faunal analyses include a sample of 7678 mammal bones collected from multiple loci and the surface layer during the 1989–1991 seasons; this sample was studied before Floor III of Brush Hut 1 was exposed [28, 46]. The assemblage was dominated by gazelle and BSGD (78%), followed by fallow deer, red deer, aurochs, wild goat, wolf, and wild cat. This sample did not include testudines and birds. No significant correlation was found between gazelle body parts and bone mineral density, as in the current study. However, a correlation was found between Thomson’s gazelle flesh units and gazelle body parts. This may show preferential transport of meat-bearing elements to the site [28]. These results differ from the ones described in this study, where we see presence of all skeletal elements, no correlation with utility indices, and a lower percentage of gazelle in the faunal assemblage. A possible explanation for the difference in results between the two studies may be the preliminary site-wide sampling, which studied bones from multiple contexts including huts, hearths, middens, and surface accumulations in the early study, as opposed to the single hut in the current one. Another possible cause is the intense and uniform fragmentation of all the bones in Brush Hut 1, regardless of species, which resulted from intense exploitation and trampling within the confines of the hut and impeded identification of possible meat-rich joints.

A detailed analysis of the fauna from Brush Hut 13 provides a comparative case, with 3779 identified specimens, of which 96% were mammals and 4% were reptiles [99]. Gazelle and BSGD bones were the most common (89%), followed by fallow deer (7%), hare (3%), and small numbers of roe deer, fox, and porcupine (<1%). The bones were extremely fragmented, especially long bone shafts, as was documented in Brush Hut 1. One obvious difference was the small number of tortoise specimens in Brush Hut 13 (NISP = 230; 6.1%) compared with Brush Hut 1 (NISP = 1729; 39.3%), along with the small number of fox bones (NISP = 8; 0.2%) in Brush Hut 13, in contrast to Brush Hut 1 (NISP = 228; 5.5%).

Based on skeletal element frequency in Brush Hut 1, it seems that the Ohalo II hunters brought back whole animals to the camp and processed them there for consumption. The animals may have been initially butchered into transportable packages, but all parts were brought back, which concurs with the findings from Brush Hut 13 [99]. The idea of limbs as the meat-rich elements being preferentially transported is not supported here. Evidence of meat consumption from ethnographic sources show extensive utilization of axial elements within the base camp [100], with the axial parts cooked and picked clean of meat, resulting in high fragmentation of these elements. The morphology of vertebrae and ribs required breaking them apart to reach all the meat. In Brush Hut 1, the high rate of fragmentation seems to indicate intensive exploitation of all nutrition-rich elements; limbs and vertebrae were completely splintered, skulls split, and phalanges halved. In the earlier Ohalo II faunal study [28, 46] phalanx splitting was reported on 28% of BSGD and BSGB 1^st^ and 2^nd^ phalanges. Split phalanges are considered a sign of intensive exploitation, but as seems to be the case in other southern Levantine Epipaleolithic sites [7], it is not only a consequence of desperation or lack of resources; even when resources are rich, all edible parts of the animal are utilized. Bone fragment sizes of both gazelle and fallow deer are remarkably homogeneous (S3 and S4 Figs in S1 File), suggesting that breakage was consistent. Preservation and/or storage of meat may also play a role in skeletal element representation [101]. Vertebrae, ribs, scapulae and pelves may be part of a butchery package ideal for drying and smoking meat, as the flesh is relatively thin in these areas [102]. Even in an area with seasonally abundant resources, food could be gathered and stored for year-round consumption.

**Fig 13:**
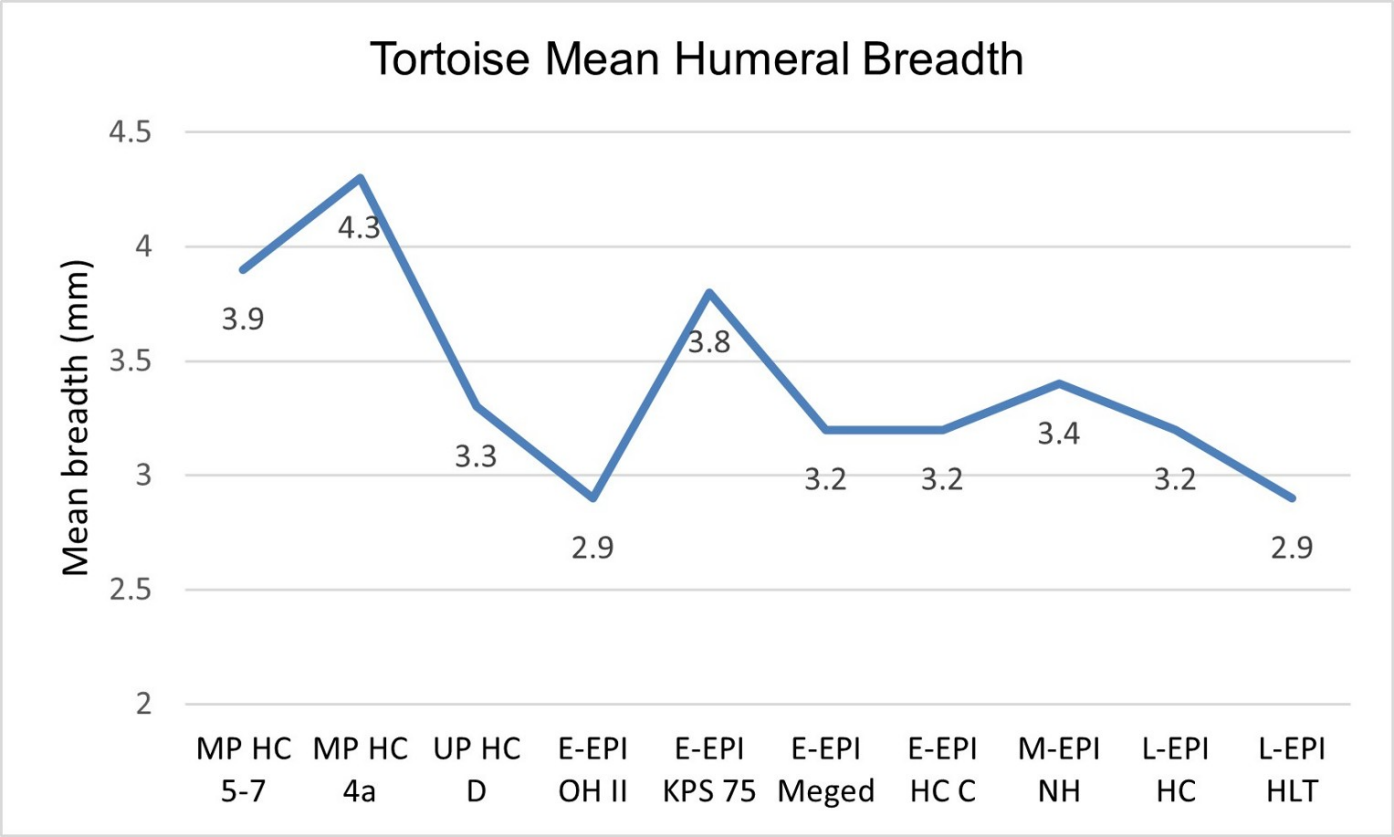
Mean humeral breadth of tortoises from Levantine sites from the middle Paleolithic through the end of the Epipaleolithic period. MP = Middle Paleolithic, UP = Upper Paleolithic, E-EPI = Early Epipaleolithic, M-EPI = Middle Epipaleolithic, L-EPI = Late Epipaleolithic. Measurements taken from medio-lateral breadth of humerus shaft at its narrowest point. Data for Hayonim Cave (HC) MP and UP from [15]; Hilazon Tachtit (HLT; n = 17) and L-EPI HC (n = 191) from [106]; Kerak Plateau sites (KPS 75; n = 10), Meged (n = 58), Nahal Hadera V (NH; n = 51), E-EPI HC (n = 63) from [105]. E-EPI OH II (n = 21). See Ohalo II Brush Hut 1 measurements in S10 Table in S1 File.

Testudines may be used as a marker of changing dietary breadth and hunting strategy during the Epipaleolithic [1, 2, 4–6]. Most of the Testudines that were found in Brush Hut 1 were tortoises, so they are used for the sake of the discussion here. At a littoral camp, where aquatic resources are extensively exploited, we see a purposeful choice to forage land-based tortoises rather than local freshwater turtles. Tortoise populations generally cannot survive annual losses of more than 4–7%, reflecting a population very sensitive to over-predation [2]; therefore, intensive exploitation will overwhelm and decimate local tortoise populations relatively quickly. Moreover, selective exploitation of large-size tortoises influences the size distribution of a population [103, 104]. The phenomenon of tortoise body-size fluctuation through time has been well-documented in the Levant and is generally interpreted as body size diminution resulting from over-hunting [1–4, 14, 15, 54, 105, 106]. The Ohalo II tortoises are small in size even relative to other Epipaleolithic assemblages (Fig 13).

Aggregated data from nine sites in the southern Levant, from the Middle Paleolithic through the Natufian periods, illustrate the different body sizes of tortoise populations. The largest tortoises are from Middle Paleolithic Hayonim Cave, the Upper Paleolithic layer shows a slight decrease in size, then the Ohalo II tortoises present much smaller specimens. Another outlier is the tortoise sample-group from KPS-75, a site on the Kerak Plateau in Jordan, where the tortoises are larger than in other Epipaleolithic sites. The authors [105] ascribe the large size to light human impact on local ecosystems. A similar line of reasoning may explain the small size of the Ohalo II tortoises- a *heavy* human impact on local tortoises, enough to affect the size of tortoises in the local populations. However, it is important to note that the maximum size of *T*. *graeca* in modern Israel varies greatly at different latitudes. In southern and central Israel, the maximum straight carapace length (SCL; measurement from the anterior edge of the carapace to the tip of posterior peripherals) of *T*. *graeca* ranges between 147 and 163 mm, and in northern Israel, between 213 and 258 mm [88]. Thus, body size is strongly correlated with a geographic north-south gradient, and the geographic impact on tortoise body-size in archaeological assemblages must be considered.

Another option is the possible preferred selection of small tortoises for use as vessels. The interpretation of the diminution in tortoise size assumes that humans will always prefer to forage large individuals since those bear more meat. However, there is evidence that tortoises were not only used as food, but that their shells were also used as containers. At Kebara Cave, Speth and Tchernov [107] suggested that the largest tortoises were used as natural “cooking pots”, with larger volume containers preferred to maximize cooking capacity. However, if carapaces were intended for use as hand-held vessels, then smaller-size adult tortoises would be the preferred target. The target range would be narrow; small enough to fit in the hand, yet adult, since the bony shells of juveniles are thin and sutures between different bone elements may not be fully closed. Most of the tortoises at Ohalo II are almost identical in size, as is evidenced by the measurement of the humeri with SD of only 0.5 mm (S10 Table in S1 File). The carapace length (as calculated based on correlation between humerus breadth and carapace length) of most Ohalo II tortoises was 90–125 mm, fitting comfortably in human palms. This standardization of size may hint at a preference for a certain size by humans. If turned upside down tortoise carapaces could be used as bowls [108]. Tortoise carapaces of similar size in the Natufian layers of Hayonim Cave and Hayonim Terrace [2, 15] were interpreted as vessels. These were produced after removing all the peripheral bones and grinding the free edges of the costal bones. Use wear modifications resulted in polish and striations on the interior of the vessel [109]. However, no signs of striations, polish or grinding was detected on the tortoise carapaces from Brush Hut 1.

The bird assemblage indicates exploitation of multiple environments and perhaps targeted usage for specific bird types. Falcons and hawks were overrepresented by feet elements in comparison with other groups, as noted previously by Simmons and Nadel [36]. Evidence for specialized use of eagle wings was also found at Wadi Jilat 22 [110]. Furthermore, the unique contexts of fox remains are noteworthy. At Ohalo II, a fox mandible was found with a long bone point associated with it [111: Fig 7, 112: Fig 5]. Fox also became important in various domestic and burial contexts, as evident at Kharaneh IV [113: Fig 7] and Uyyun al-Hammam [114; and see 115 for Natufian examples and further discussion]. The presence of abundant fox and hare feet elements may suggest use of their pelts [116].

This study found that bone colors ranging from orange to brown to black in a water-logged site cannot be definitively correlated with burning or exposure to high temperatures. The bones may have been exposed to lower cooking temperatures, but this cannot be differentiated from other diagenetic processes. The grey and white bones displayed a crystal structure consistent with very high intensity burning at temperatures above 500°C. Bones used as fuel or disposed of in a hearth may reach this level of crystallinity. Why the splitting factor is substantially higher than that of burnt/diagenetic bones from other archaeological sites is a question that requires further analysis; however, given that FTIR spectra were obtained mainly from dry cave sites, the depositional environment probably played an important role here. The grey and white bones were intensively burnt, and then remained in a water-logged depositional environment. The Ohalo II lake water was a relatively brackish environment with a high pH level (between 8.1–7.4) [23], which may lead to recrystallization of bone apatite [117]. Additionally, a post-depositional increase in the salinity of the archaeological layer [23] may have led to the unusually high crystallinity of the burnt bones. A sample of fish bones from Brush Hut 1 examined with FTIR agrees with the results of this study—only grey and white bones could be definitively interpreted as burned (<1% of fish bones in the hut) [30], while dark brown and black colored bones were the result of mineral staining [30, 118].

The spatial distribution of bones and plants indicated the presence of activity areas. On Floor III, most of the bones were found around and in the ashes of the central hearth. On Floor II, the main concentration of bones was in the southern part of the hut, while cereal grains were concentrated in the northern part of the hut, around the grinding stone. Snir et al. [29] suggested that Floor III represents a winter occupation based on the plant assemblage, the grass bedding, and the presence of an indoor hearth. The mesowear analysis of gazelle teeth from Floor III supported this hypothesis. We may see exploitation of plant-based vs. animal-based foods changing seasonally, when a decrease in the availability of one resource is balanced by a rise in the exploitation of the other.

The botanical macro remains from Brush Hut 1 support an occupation during the spring and early summer [10]. The bird species are found throughout the year, with a peak during autumn–winter (October–March; [36]). The numerous fish remains attest to fishing according to breeding season, cichlids in spring and summer (April-September) and the cyprinids in winter and spring (January-April; [30]). The presence of gazelle fetal bones, and an indoor hearth suggest a winter occupation on Floor III. The relatively high percentage of fragmentation that was observed in the NISP:MNE ratio of bones on Floor III may be attributed to trampling or to intensive processing of bone for marrow and grease extraction [101, 119]. The discrepancy between tooth microwear and mesowear results and the low variability in the number of scratches on teeth, both indicate a short-term seasonal signal; i.e. gazelle and fallow deer were killed and brought back to the site during seasonal events spanning about three months, from winter to early spring. The microwear pattern of the gazelle teeth from Brush Hut 1 fits with the pattern of winter to early spring mortality, supporting the results obtained from analysis of the bird assemblage. Brush Hut 1 was probably occupied throughout the year, with exploitation of seasonally-available resources.

### Comparison with other Levantine Early Epipaleolithic sites

Comparison of the Brush Hut 1 assemblage with other Levantine Early Epipaleolithic sites (Fig 1) showed a general similarity in species richness (ungulates, fox, hare, tortoise), with the difference manifested in species evenness (Table 8). For example, the site of Nahal Hadera V (ca. 21,300–18,000 BP), located on the Mediterranean coast, has a faunal assemblage with about 95% ungulates (fallow deer and gazelle) and <5% small game (fox, hare, birds, tortoise) [120, 121]. The Kebaran assemblage from Meged Rockshelter, located in the Mediterranean ecozone (western Galilee), consists of 61% ungulates, 38% small game and 1% carnivores [15]. The nearby Hayonim C Kebaran faunal assemblage encompasses 80% ungulates, 19% small game and 1% carnivores [15, 46]. Nahal Ein Gev I was an open-air early Kebaran site, located near the eastern shore of the Sea of Galilee. The faunal assemblage consists of 80% ungulates, 5% fox, 7% hare and 6% tortoise. No fish bones were reported [122].

**Table 8:** Early Epipaleolithic sites and the percentage of ungulates and small game within their faunal assemblages.

Geographical Location	Site	Date (BP)	Ungulates %	Small game %	References
Mediterranean Coast	Nahal Hadera V	21,300–18,000	95	<5	[120, 121]
Western Galilee	Meged Rockshelter	22,000–18,000	61	38	[15]
Western Galilee	Hayonim C	17,000–14,000	80	19	[15, 46]
Upper Jordan Valley	Ein Gev I	Kebaran (21,000–17,000)	80	18	[122]
Lower Jordan Valley	Urkan e-Rubb II	18,200–17,200	95	5	[123]
Azraq Basin	Kharaneh IV	20,000–18,000	91	7	[124, 125]
Jordanian Highlands	KPS-75, Tor at-Tareeq, Tor Sageer, Yutil-al Hasa	25,000–18,500	40–70	30–60	[105]

Small game includes fox, hare, birds and tortoises. Map of sites in Fig 1.

Urkan e-Rubb IIa, located in the arid portion of the Lower Jordan Valley, was a seasonal site with an economy focused on gazelle hunting (90%), with low numbers of additional ungulates, carnivores, and small game [123]. East of the Jordan Valley in the Azraq Basin, the phase A faunal assemblage of Kharaneh IV (ca. 20,000–18,000 cal BP) is comprised of 91% ungulates [124]. Though located in a generally arid area, there is evidence of a large wetland during the time of occupation [125]. A study of four contemporaneous sites (ca. 25,000–18,500 cal BP) in the western Jordanian highlands showed that hunting of prime-ranked gazelles accounts for 40–70% of the assemblages while small game, mainly tortoise, the other 30–60% [105]. The sites were occupied during a time when the highlands were relatively well-watered.

Compared with most of the above sites, ungulates are less abundant in Brush Hut 1. Ungulates compose 44% of the NISP in Brush Hut 1, as opposed to over 50% and up to 95% in the sites cited above. However, Brush Hut 1 had a much higher incidence of small animal exploitation, with 48% of the assemblage composed of small mammals and Testudines, an additional 6% of birds, and a very high number of fish. Within the site itself, Brush Hut 13 presents a very different assemblage, dominated by gazelles and with very few foxes or tortoises. When the tortoise and bird assemblages were excluded for the sake of discussion, gazelles became 57% of the Brush Hut 1 assemblage and fallow deer 21%. Yet, 20% of the assemblage consisted of small mammals (fox, hare, hedgehog) which is unusual for sites of this period. When examining by floor, Floor II contained 42% ungulates and Floor III 52% ungulates. Gazelle and fallow deer represented the bulk of the meat, with easily foraged tortoises also widely taken. Tortoises did not replace larger ungulates as a food source but were foraged as a supplement, as well as possibly for use as vessels. Furthermore, the role of fish in the diet was significant [30].

It was suggested that during the Late Epipaleolithic (Natufian) period, gazelles were hunted in a specialized manner which led to changes in the gazelle bone assemblages, such as increase in young gazelles [126], a higher proportion of larger males [127] and possibly general diminution in body size [127, 128]. Not all assemblages conform to this hypothesis [121], showcasing the high variability of gazelle assemblages in the southern Levant. We compared gazelle metapodial measurements from Brush Hut 1 (S11 Table in S1 File) with a range of Epipaleolithic sites with published measurements—Nahal Hadera V, Hefzibah, Neve David, and el-Wad Terrace in the Mediterranean zone [121], and Urkan-e-Rubb IIa in the arid Jordan Valley [123]—to assess how the Ohalo II gazelles fit into the Epipaleolithic sequence. The results indicated that the Ohalo II gazelle body size was similar to, or very slightly larger than, other gazelle populations. Uniformity of body size through the Epipaleolithic sequence may suggest low hunting pressure.

### Brush Hut 1 and the NCT and OFT models

The Ohalo II lake shore presents a prime location, with access to fresh water, fish, birds, mammals, and a range of edible plants. Favourable environmental conditions are reflected in the abundant cereal and plant remains from Ohalo II [27, 29, 38]. Phytolith evidence indicates incorporation of plants from steppe, woodland and wetland environments [129]. Paleoenvironmental reconstruction from palynological records indicated no significant reduction in humidity, and the presence of open park forest during the LGM in the Ohalo II vicinity [130, 131]. Analysis of stable carbon isotopes (δ^13^C) from the Zalmon cave showed increased humidity in the northern Jordan Valley during the LGM [132]. Faunal species diversity and evenness within Brush Hut 1 do not indicate an ungulate-focused diet; rather, the Ohalo II hunters exploited a wide niche of prey animals available in the rich local habitats.

Referring to the general criteria for possible faunal signatures of the OFT or the NCT, as outlined in Table 1, the Brush Hut 1 faunal assemblage mainly indicates a setting of resource abundance in accordance with the NCT model. Evidence of resource abundance includes exploitation of both high-ranking larger ungulates and high-ranking slow game. Another sign for abundance is found in the ratio of female (55%) to male (45%) gazelles, reflecting contemporary adult herd structure whereby there are often more females than males [133], which may signal hunting of the locally available gazelle herds without needing to seek larger males. Demographic data also indicate hunting of mainly prime-aged adults. The severe breakage of all faunal components in Brush Hut 1, and the presence of percussion and cut marks on some bones, indicates intensive processing which may be found according to either model.

The OFT would predict resource depression and a decrease in large game and Testudines over time (from Floor III to I), yet in Brush Hut 1 there is an increase in ungulates from Floor III to I. Small, quick game are best represented on Floor III, with a much lower frequency on Floor II. Testudines form a large percentage of the assemblage on both Floors III and II (Table 4), and their body-size remained steady through repeated occupations. There is also clear evidence for the exploitation of diverse aquatic resources, birds, and abundant high-quality plant resources. The comparison with contemporaneous Levantine sites (Table 8) seems to indicate that the Ohalo II occupants pursued a significantly more varied diet compared to other sites, even when compared to those located in the Mediterranean zone. However, in light of lower preservation conditions in other sites this picture may be biased.

It seems there was no "forced" exploitation of lower-ranked prey as explained by OFT. The Ohalo II hunters preferred adult ungulates; there was no need to expend extra energy in hunting quick, difficult to catch hares, for example, when large quantities of fish, birds, turtles, and plants were accessible. The choice of a littoral habitat that could be intensely exploited is an example of niche selection. Fishing was possibly a year-round activity, and a rich source of nutrition with a low cost of acquisition [30, 37]. It does not seem that climatic pressure and demographic forcing were factors driving the composition of the Brush Hut 1 faunal assemblage. Availability of a large variety of local prey prevented heavy dependence on one or two prey types.

## Conclusions

Brush Hut 1 at Ohalo II presents a different picture of subsistence than most other early Epipaleolithic sites. Climatic oscillations during the LGM had minimal effects on the Upper Jordan Valley, specifically in the vicinity of Ohalo II, enabling the Ohalo II people to utilize a broad ecological niche comprised of varied edible plants, mammals, reptiles, birds, and fish. Broad spectrum foraging is not necessarily an indicator of stress in this case; rather, it may be an indicator of plenty. Despite the ability to hunt prime adult ungulates, a wide range of prey was exploited. Extreme fragmentation of an assemblage is not necessarily an indicator of subsistence stress; even in settings with abundant resources, carcasses could be fully utilized down to the marrow. Tortoises were seemingly selected for a specific body-size, which may suggest that their shells, and not their meat, were the main target. Hare and fox were possibly hunted for their pelts. FTIR analysis shows that bone color in water-logged sites may to a certain degree indicate burning, although the colors are different than those of bones from dry sites. The chemical composition of some of the different colored/stained bones indicate intensive diagenetic processes due to the depositional environment of Brush Hut 1 and not to burning. Floors III and II reflect different organization of space; Floor III is the earliest and the most heavily used, with the broadest range of species. Ohalo II presents a unique opportunity to test the contrasting models of NCT and OFT for resource diversification; the Brush Hut 1 evidence points to a case of resource abundance through careful niche selection where the exploitation of a broad array of predictable resources was possible. This best mirrors the NCT model in many ways, though certainly preservation plays an important role in recognizing the varied resources exploited in the past. Finally, Ohalo II reflects a true broad-spectrum economy during the LGM at the very beginning of the Epipaleolithic period.

## Supporting information

S1 File (DOCX)
