# Supplementary material for: Abundance or stress? Faunal exploitation patterns and subsistence strategies: The case study of Brush Hut 1 at Ohalo II, a submerged 23,000-year-old camp in the Sea of Galilee, Israel
Source: PLoS One. 2022 Jan 26;17(1):e0262434. doi: 10.1371/journal.pone.0262434 (PMC8791512; doi:10.1371/journal.pone.0262434)
Supplement: S1 File — (DOCX) [file pone.0262434.s001.docx]

**Supporting Information**

**S1 Table. Gazelle - all identified skeletal elements (S.E.) (NISP = 399).**

| *Gazella gazella* |  |  |  |  |  |  |  |
| --- | --- | --- | --- | --- | --- | --- | --- |
| S.E. | **NISP** | **MNE** | **%MAU** | **S.E** | **NISP** | **MNE** | **%MAU** |
| SKULL | 9 | 3 | 35.29 | FEMUR P | 7 | 7 | 41.18 |
| MANDIBLE | 15 | 4 | 23.53 | FEMUR D | 5 | 5 | 29.41 |
| HORNCORE | 8 | 8 | 47.06 | FEMUR SH | 4 | 3 | 17.65 |
| ATLAS | 3 | 3 | 35.29 | TIBIA P |  |  | 0.00 |
| AXIS | 1 | 1 | 11.76 | TIBIA D | 9 | 9 | 52.94 |
| CERVICAL | 2 | 2 | 4.71 | TIBIA SH | 4 | 2 | 11.76 |
| THORACIC | 9 | 6 | 5.41 | MTT PROX | 8 | 7 | 41.18 |
| LUMBAR | 12 | 4 | 6.71 | MTT DIST | 1 | 1 | 5.88 |
| SACRUM |  |  | 0.00 | MTT SH |  |  | 0.00 |
| CAUDAL |  |  | 0.00 | MTP P | 1 | 1 | 5.88 |
| RIB | 26 | 21 | 9.41 | MTP DIST | 17 | 17 | 100.00 |
| SCAPULA | 3 | 2 | 11.76 | MTP SH | 3 | 1 | 5.88 |
| HUMERUS P | 4 | 4 | 23.53 | ELC | 12 | 12 | 70.59 |
| HUMERUS D | 10 | 10 | 58.82 | MAG | 12 | 12 | 70.59 |
| HUMERUS SH | 1 | 1 | 5.88 | LUNATE |  |  | 0.00 |
| RADIUS P | 4 | 4 | 23.53 | SCAPH | 7 | 7 | 41.18 |
| RADIUS D | 7 | 7 | 41.18 | UNCI | 10 | 10 | 58.82 |
| RADIUS SH | 5 | 2 | 11.76 | CUNE | 4 | 4 | 23.53 |
| ULNA P | 1 | 1 | 5.88 | LM | 3 | 3 | 17.65 |
| ULNA D | 1 | 1 | 5.88 | PISI |  |  | 0.00 |
| ULNA SH |  |  | 0.00 | NC | 7 | 7 | 41.18 |
| MTC P | 1 | 1 | 5.88 | CALCANEUM | 11 | 7 | 41.18 |
| MTC D | 1 | 1 | 5.88 | ASTRAGALUS | 9 | 9 | 52.94 |
| MTC SH | 2 | 2 | 11.76 | PH1 | 37 | 24 | 35.29 |
| ACETABULUM | 9 | 6 | 35.29 | PH2 | 45 | 33 | 48.47 |
| ISCHIUM | 1 | 1 | 5.88 | PH3 | 47 | 37 | 54.35 |
| ILIUM | 1 | 1 | 5.88 | PATELLA | 6 | 4 | 23.53 |
| PUBIS |  |  | 0.00 |  |  |  |  |

| BSGD |  |  |  |  |  |  |  |
| --- | --- | --- | --- | --- | --- | --- | --- |
| S.E. | **NISP** | **MNE** | **%MAU** | **S.E.** | **NISP** | **MNE** | **%MAU** |
| SKULL | 82 | 6 | 100.00 | FEMUR P | 6 | 6 | 50.00 |
| MANDIBLE | 9 | 4 | 33.33 | FEMUR D | 1 | 1 | 8.33 |
| HORNCORE/ANTLER | 4 | 4 | 33.33 | FEMUR SH | 1 | 1 | 8.33 |
| ATLAS |  | 0 | 0.00 | TIBIA P | 2 | 2 | 16.67 |
| AXIS | 3 | 2 | 33.33 | TIBIA D | 1 | 1 | 8.33 |
| CERVICAL | 17 | 5 | 16.67 | TIBIA SH | 5 | 2 | 16.67 |
| THORACIC | 6 | 6 | 7.50 | MTT PROX | 2 | 2 | 16.67 |
| LUMBAR | 19 | 7 | 16.50 | MTT DIST |  | 0 | 0.00 |
| SACRUM | 1 | 1 | 16.67 | MTT SH | 3 | 2 | 16.67 |
| CAUDAL | 2 | 1 | 0.00 | MTP P | 15 | 15 | 62.50 |
| STERNUM | 1 | 1 | 16.67 | MTP DIST | 59 | 13 | 54.17 |
| RIB | 112 | 9 | 5.33 | MTP SH | 15 | 3 | 12.50 |
| SCAPULA | 18 | 4 | 33.33 | ELC | 1 | 1 | 8.33 |
| HUMERUS P | 2 | 2 | 25.00 | MAG | 2 | 2 | 16.67 |
| HUMERUS D | 7 | 7 | 66.67 | LUNATE |  | 0 | 0.00 |
| HUMERUS SH | 1 | 1 | 8.33 | SCAPH |  | 0 | 0.00 |
| RADIUS P | 2 | 2 | 16.67 | UNCI |  | 0 | 0.00 |
| RADIUS D | 2 | 2 | 16.67 | CUNE | 5 | 5 | 41.67 |
| RADIUS SH | 5 | 2 | 16.67 | LM |  | 0 | 0.00 |
| ULNA P | 2 | 2 | 16.67 | PISI | 1 | 2 | 16.67 |
| ULNA D | 3 | 2 | 16.67 | NC | 2 | 2 | 16.67 |
| ULNA SH | 1 | 1 | 8.33 | CALCANEUM | 7 | 7 | 58.33 |
| MTC P | 2 | 2 | 16.67 | ASTRAGALUS | 1 | 1 | 8.33 |
| MTC D |  | 0 | 0.00 | PH1 | 28 | 20 | 41.58 |
| MTC SH | 1 | 1 | 8.33 | PH2 | 41 | 31 | 64.50 |
| ACETABULUM | 6 | 3 | 25.00 | PH3 | 15 | 10 | 20.75 |
| ISCHIUM | 2 | 2 | 16.67 | PATELLA | 3 | 2 | 16.67 |
| ILIUM |  | 0 | 0.00 |  |  |  |  |
| PUBIS |  | 0 | 0.00 |  |  |  |  |

**S2 Table. BSGD - all identified skeletal elements (NISP = 529).**

**S3 Table. Abbreviations used in graphs and tables throughout the text.**

| **Skeletal Elements- Abbreviations** | |  |  |
| --- | --- | --- | --- |
| **ANT** | Antler | **NC** | Naviculo-cuboid |
| **CALC** | Calcaneum | **PH** | Phalanx |
| **CUN** | Cuneiform | **PISI** | Pisiforme |
| **ELC** | External lateral cuneiform | **RAD** | Radius |
| **FEM** | Femur | **SCAPH** | Scaphoid |
| **HUM** | Humerus | **SK** | Skull |
| **LM** | Lateral Malleolus | **TIB** | Tibia |
| **MAND** | Mandible | **UNCI** | Unciform |
| **MAG** | Magnum | **VERT** | Vertebrae |
| **MTC** | Metacarpal |  |  |
| **MTP** | Metapodials |  |  |
| **MTT** | Metatarsal |  |  |

| **Skeletal Portions** | |
| --- | --- |
| **P** | Proximal |
| **D** | Distal |
| **SH** | Shaft |
| **U** | Upper |
| **L** | Lower |
| **LB** | Long bone |
| **FR** | Fragment |

**S4 Table. Fallow deer - all identified skeletal elements (NISP = 146).**

| *D. mesopotamica* |  |  |  |  |  |  |  |
| --- | --- | --- | --- | --- | --- | --- | --- |
| S.E. | **NISP** | **MNE** | **%MAU** | **S.E.** | **NISP** | **MNE** | **%MAU** |
| SKULL |  |  |  | FEMUR P | 2 | 2 | 20.0 |
| MANDIBLE | 18 | 10 | 100.0 | FEMUR D | 2 | 2 | 20.0 |
| HORNCORE/ANTLER |  |  |  | FEMUR SH |  |  |  |
| ATLAS |  |  |  | TIBIA P | 3 | 3 | 30.0 |
| AXIS | 2 | 2 | 40.0 | TIBIA D | 4 | 4 | 40.0 |
| CERVICAL | 3 | 2 | 8.0 | TIBIA SH | 3 | 2 | 20.0 |
| THORACIC | 9 | 5 | 7.4 | MTT PROX |  |  |  |
| LUMBAR | 11 | 4 | 11.2 | MTT DIST | 1 | 1 | 10.0 |
| SACRUM |  |  |  | MTT SH | 7 | 4 | 40.0 |
| CAUDAL |  |  |  | MTP P |  |  |  |
| RIB | 16 | 9 | 6.4 | MTP DIST | 5 | 5 | 50.0 |
| SCAPULA | 2 | 2 | 20.0 | MTP SH | 4 | 2 | 20.0 |
| HUMERUS P | 1 | 1 | 10.0 | ELC | 1 | 1 | 10.0 |
| HUMERUS D | 4 | 4 | 40.0 | MAG | 2 | 2 | 20.0 |
| HUMERUS SH | 1 | 1 | 10.0 | LUNATE | 1 | 1 | 10.0 |
| RADIUS P | 3 | 3 | 30.0 | SCAPH | 1 | 1 | 10.0 |
| RADIUS D | 2 | 2 | 20.0 | UNCI | 1 | 1 | 10.0 |
| RADIUS SH | 1 | 1 | 10.0 | CUNE |  |  |  |
| ULNA P | 3 | 3 | 30.0 | LM |  |  |  |
| ULNA D | 1 | 1 | 10.0 | PISI |  |  |  |
| ULNA SH |  |  |  | NC | 2 | 2 | 20.0 |
| MTC P | 1 | 1 | 10.0 | CALCANEUM | 1 | 1 | 10.0 |
| MTC D | 1 | 1 | 10.0 | ASTRAGALUS |  |  |  |
| MTC SH | 2 | 2 | 20.0 | PH1 | 10 | 7 | 17.5 |
| ACETABULUM |  |  |  | PH2 | 10 | 7 | 17.5 |
| ISCHIUM |  |  |  | PH3 | 3 | 3 | 7.5 |
| ILIUM | 1 | 1 | 10.0 | PATELLA | 1 | 1 | 10.0 |
| PUBIS |  |  |  | STERNUM | 1 | 1 | 20.0 |

**S5 Table. BSGB - all identified skeletal elements (NISP = 146).**

| BSGB |  |  |  |  |  |  |  |
| --- | --- | --- | --- | --- | --- | --- | --- |
| S.E. | **NISP** | **MNE** | **%tMAU** | **S.E.** | **NISP** | **MNE** | **%MAU** |
| SKULL | 11 | 3 | 100.00 | FEMUR P | 3 | 3 | 50.00 |
| MANDIBLE | 1 | 1 | 16.67 | FEMUR D | 4 | 4 | 66.67 |
| HORNCORE/ANTLER | 82 | 3 | 50.00 | FEMUR SH | 9 | 3 | 50.00 |
| ATLAS |  | 0 | 0.00 | TIBIA P |  | 0 | 0.00 |
| AXIS | 1 | 1 | 33.33 | TIBIA D | 3 | 3 | 50.00 |
| CERVICAL | 12 | 4 | 26.67 | TIBIA SH | 3 | 2 | 33.33 |
| THORACIC | 3 | 2 | 5.00 | MTT PROX | | 0 | 0.00 |
| LUMBAR | 8 | 2 | 9.33 | MTT DIST |  | 0 | 0.00 |
| SACRUM |  | 0 | 0.00 | MTT SH | 4 | 2 | 33.33 |
| CAUDAL |  | 0 | 0.00 | MTP P | 1 | 1 | 16.67 |
| RIB | 33 | 7 | 8.33 | MTP DIST | 5 | 5 | 83.33 |
| SCAPULA | 6 | 2 | 33.33 | MTP SH | 6 | 2 | 33.33 |
| HUMERUS P | | 0 | 0.00 | ELC |  | 0 | 0.00 |
| HUMERUS D | 3 | 3 | 50.00 | MAG | 1 | 1 | 16.67 |
| HUMERUS SH | 1 | 1 | 16.67 | LUNATE |  | 0 | 0.00 |
| RADIUS P |  | 0 | 0.00 | SCAPH | 2 | 1 | 16.67 |
| RADIUS D | 1 | 1 | 16.67 | UNCI |  | 0 | 0.00 |
| RADIUS SH | 5 | 2 | 33.33 | CUNE |  | 0 | 0.00 |
| ULNA P |  | 0 | 0.00 | LM |  | 0 | 0.00 |
| ULNA D | 1 | 1 | 16.67 | PISI | 4 | 1 | 16.67 |
| ULNA SH |  | 0 | 0.00 | NC | 1 | 1 | 16.67 |
| MTC P |  | 0 | 0.00 | CALCANEUM | 2 | 2 | 33.33 |
| MTC D |  | 0 | 0.00 | ASTRAGALUS | | 0 | 0.00 |
| MTC SH |  | 0 | 0.00 | PH1 | 17 | 12 | 50.00 |
| ACETABULUM | 2 | 1 | 16.67 | PH2 | 18 | 12 | 45.83 |
| ISCHIUM | 1 | 1 | 16.67 | PH3 | 2 | 2 | 8.33 |
| ILIUM | 1 | 1 | 16.67 | PATELLA |  | 0 | 0.00 |
| PUBIS | 1 | 1 | 16.67 | STERNUM | | 0 | 0.00 |

**S6 Table. Fox - all identified skeletal elements (NISP = 197).**

| S.E | NISP | MNE | %MAU | S.E. | NISP | MNE | %MAU |
| --- | --- | --- | --- | --- | --- | --- | --- |
| SKULL | 1 | 1 | 25.0 | ACETABULUM | 1 | 1 | 12.5 |
| MANDIBLE | 9 | 8 | 100.0 | ISCHIUM |  |  |  |
| ATLAS | 2 | 2 | 50.0 | PUBIS |  |  |  |
| AXIS |  |  |  | FEMUR P | 4 | 4 | 50.0 |
| CERVICAL |  |  |  | FEMUR D | 1 | 1 | 12.5 |
| THORACIC |  |  |  | FEMUR SH | 1 | 1 | 12.5 |
| LUMBAR |  |  |  | TIBIA P | 1 | 1 | 12.5 |
| SACRUM |  |  |  | TIBIA D | 2 | 2 | 25.0 |
| CAUDAL | 3 | 3 | 5.3 | TIBIA SH |  |  |  |
| RIB (26) | 17 | 9 | 8.5 | MTT P | 4 | 5 | 12.5 |
| SCAPULA | 1 | 1 | 12.5 | MTT D |  |  |  |
| HUM P | 1 | 1 | 12.5 | MTT SH | 1 | 1 | 12.5 |
| HUM D | 2 | 2 | 25.0 | MTP P | 4 | 4 | 10.0 |
| HUM SH | 1 | 1 | 12.5 | MTP D | 17 | 17 | 42.5 |
| RADIUS P | 6 | 6 | 75.0 | MTP SH | 1 | 1 | 12.5 |
| RADIUS D | 4 | 4 | 50.0 | CARPAL | 11 | 11 | 19.5 |
| RADIUS SH | 1 | 1 | 12.5 | TARSALS | 5 | 5 | 12.5 |
| ULNA P | 4 | 4 | 50.0 | CALCANEUM | 2 | 2 | 25.0 |
| ULNA D |  |  |  | ASTRAGALUS | 3 | 2 | 25.0 |
| ULNA SH |  |  |  | PH1 | 9 | 9 | 11.3 |
| MTC | 12 | 11 | 27.5 | PH2 | 11 | 11 | 17.0 |
| MTC P | 3 | 3 | 7.5 | PH3 | 20 | 22 | 27.5 |
| MTC D |  |  |  | PH | 32 | 22 | 9.5 |

**S7 Table. Measurements of *Testudo graeca* specimens at the National Natural History Comparative Collections, Hebrew University, Jerusalem (NNHC-HUJI). CL = carapace length; CW = carapace width; PL = length of plastron; Humerus measurements are the medio-lateral breadth of humerus shaft at its narrowest point (mm).**

| Specimen Number | CL | CW | PL | Humerus- Left | Humerus- Right |
| --- | --- | --- | --- | --- | --- |
| Z-374 | 155 | 116 | 13.3 | 4.17 | 4.11 |
| Z-373 | 160 | 115 |  | 4.00 | 4.03 |
| Z-126 | 164 | 120 |  | 4.92 |  |
| Z-377 | 114 | 89 |  | 3.50 | 3.70 |
| Z-473 | 114 | 83 |  | 3.36 |  |
| Z-256 | 175 |  |  | 4.27 | 4.48 |
| Z-264 | 183 | 135 |  | 4.96 | 4.98 |
| Z-125 | 128 | 96 |  | 3.50 | 3.60 |
| Z-348 | 118 | 91 |  | 3.26 |  |
| Z-338 | 63.5 | 52 |  | 1.64 | 1.65 |
| Z-493 | 132 | 97 |  | 3.42 |  |

**S1 Fig. Modified General Utility Index (Binford 1978), gazelle and BSGD.** Calculated based on %MAU values from S1 and S2 Tables.

**S2 Fig. Fallow deer and BSGB utility index curves, based on normed utility indices for caribou, all floors (Binford 1978; Lyman 1994). Calculated based on %MAU values from S4 and S5 Tables.**

**S3 Fig. The %survivorship of gazelle and BSGD specimens based on bone density values (Lyman 1984, 1994; Lam et al. 1999) (NISP=504).**

**S4 Fig. Length and width measurements of all of gazelle and BSGD bones on each floor.** NISP: Floor I = 153, Floor II = 347, Floor III = 815.


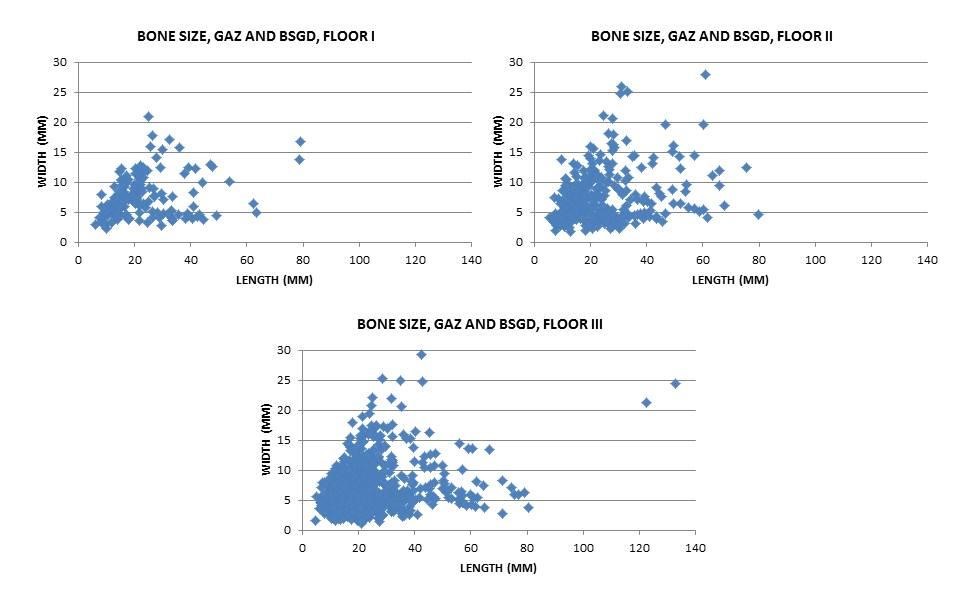


**S5 Fig. Length and width measurements of all fallow deer and BSGB bones on each floor (NISP: Floor I = 38, Floor II = 147, Floor III = 303).**

**
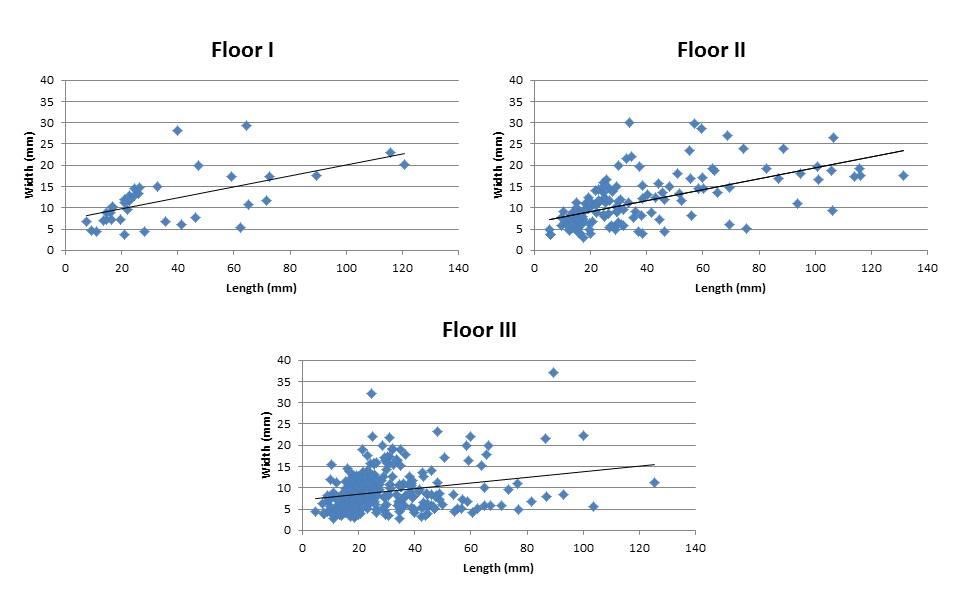
**

**S6 Fig. Illustration of T*estudo graeca* carapace with missing peripherals marked in red. Image modified after Fig. 2 in Amiranashvili 2000.**

**
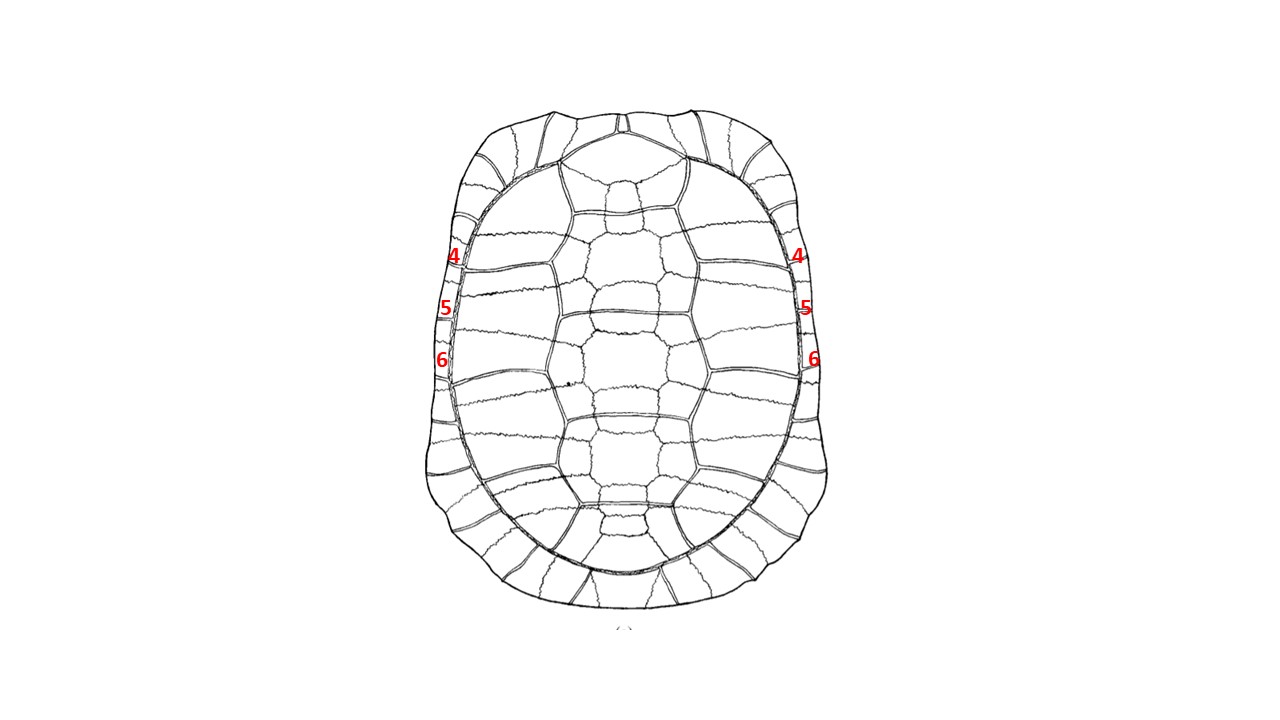
**

**S8 Table. Epiphyseal fusion for gazelle and BSGD (NISP = 274)**, all floors of Locus I. Age data (in months) from Munro et al. 2009.

| FUSION | Floor I |  |  | Floor II |  |  | Floor III |  |  |
| --- | --- | --- | --- | --- | --- | --- | --- | --- | --- |
|  | FUSED | UNF | AGE | FUSED | UNF | AGE | FUSED | UNF | AGE |
| RIB | 7 |  | N/A | 4 | 1 | N/A | 16 |  | N/A |
| HUM P |  |  |  | 1 |  | 18+ | 3 | 1 | 0-18 |
| HUM D | 1 |  | 3+ | 1 |  | 3+ | 14 |  | 3+ |
| RAD P | 1 |  | 3+ |  |  |  | 4 | 1 | 0-7 |
| RAD D | 1 |  | 18+ | 4 |  | 18+ | 2 | 2 | 0-18 |
| ULNA P |  |  |  | 1 |  | 18+ | 2 |  | 18+ |
| ULNA D | 1 |  | 18+ |  |  |  | 3 |  | 18+ |
| MTC D |  |  |  | 1 |  | 18+ |  |  |  |
| FEM P |  |  |  |  | 1 | 0-18 | 9 | 3 | 0-18 |
| FEM D | 1 |  | 18+ | 2 |  | 18+ | 3 |  | 18+ |
| TIB P |  |  |  | 1 |  | 18+ | 1 |  | 18+ |
| TIB D | 2 |  | 7+ |  | 1 | 0-18 | 3 | 3 | 0-18 |
| MTT D |  |  |  | 1 |  | 18+ |  |  |  |
| MTP D | 8 |  | 18+ | 12 |  | 18+ | 41 | 9 | 0-18 |
| CALC | 3 | 1 | 0-18 | 2 |  | 18+ | 5 | 2 | 0-18 |
| P1 P | 2 |  | 3+ | 6 |  |  | 8 | 2 | 0-7 |
| P2 P | 2 |  | 3+ | 3 |  |  | 5 | 5 | 0-7 |

**S9 Table. Raw mesowear and microwear data for the gazelle and fallow deer** from Floor III of Brush Hut 1, Ohalo II. Abbreviations: MWS = Mesowear score; NP = Average number of pits; NS = Average number of scratches; %LP = Percentage of individuals with large pits; %G = Percentage of individuals with gouges; SWS = Scratch width score; %XS = Percentage of individuals with cross scratches. (UP – upper premolar, UM – upper molar, LP – lower premolar, LM – lower molar).

| Specimen # | Species | Tooth | Side | MWS | NP | NS | LP | G | SWS | XS |
| --- | --- | --- | --- | --- | --- | --- | --- | --- | --- | --- |
| 121 | *D. mesopotamica* | UM1 | L | 2 | 34.0 | 13.0 | 1 | 1 | 1 | 0 |
| 122 | *D. mesopotamica* | UM2 | L |  | 29.0 | 13.0 | 1 | 1 | 1 | 0 |
| 124 | *D. mesopotamica* | UP3 | L | 3 | 39.5 | 12.5 | 1 | 1 | 2 | 0 |
| 130 | *D. mesopotamica* | UM1/2 | L |  | 34.5 | 12.0 | 0 | 0 | 1 | 0 |
| 174 | *D. mesopotamica* | UM |  |  | 38.5 | 12.0 | 1 | 0 | 1 | 0 |
| 197 | *D. mesopotamica* | LM1 | L | 3 | 41.5 | 11.5 | 1 | 1 | 1 | 0 |
| 196 | *D. mesopotamica* | LP3 | L |  | 44.0 | 13.5 | 1 | 1 | 1 | 0 |
| 208 | *D. mesopotamica* | UP4 |  |  | 36.5 | 12.5 | 1 | 1 | 1 | 0 |
| 100 | *G. gazella* | UM3 | R |  | 25.0 | 16.5 | 1 | 1 | 1 | 0 |
| 101 | *G. gazella* | UM2 | R | 1 | 23.0 | 18.0 | 0 | 0 | 2 | 0 |
| 104 | *G. gazella* | LM2 | R | 2 | 21.0 | 18.0 | 1 | 0 | 1 | 0 |
| 106 | *G. gazella* | UP3/4 | L | 3 | 25.5 | 20.0 | 0 | 0 | 1 | 0 |
| 107 | *G. gazella* | UM2 | R | 0 | 21.0 | 22.5 | 1 | 0 | 1 | 0 |
| 110 | *G. gazella* | LM3 | L | 1 | 33.5 | 19.5 | 1 | 0 | 1 | 0 |
| 111 | *G. gazella* | UM1 | R | 2 | 22.0 | 20.5 | 1 | 0 | 1 | 0 |
| 114 | *G. gazella* | LM3 | L | 2 | 18.5 | 19.0 | 0 | 0 | 0 | 0 |
| 118 | *G. gazella* | UM3 | L |  | 27.0 | 15.5 | 1 | 1 | 2 | 0 |
| 119 | *G. gazella* | LM1 | L | 4 | 33.0 | 22.0 | 0 | 0 | 1 | 0 |
| 126 | *G. gazella* | UM3 | R |  | 20.5 | 18.5 | 1 | 1 | 2 | 0 |
| 162 | *G. gazella* | LM |  | 1 | 18.0 | 23.5 | 0 | 0 | 1 | 0 |
| 202 | *G. gazella* | UM3 | L | 1 | 20.5 | 17.0 | 0 | 0 | 1 | 0 |

**S10 Table. *Testudo graeca* humerus measurements from Brush Hut 1.** Measurements (mm) of medio-lateral breadth of humerus shaft at its narrowest point.

| Specimen number | Humerus (mm) |
| --- | --- |
| 9937 | 3.31 |
| 9938 | 3.30 |
| 9940 | 3.57 |
| 9941 | 3.30 |
| 9942 | 3.10 |
| 9945 | 3.41 |
| 9946 | 2.85 |
| 9948 | 3.05 |
| 9952 | 1.82 |
| 9956 | 2.86 |
| 9959 | 2.73 |
| 9964 | 2.31 |
| 9968 | 3.44 |
| 9971 | 3.08 |
| 9973 | 2.98 |
| 9987 | 3.55 |
| 9988 | 2.88 |
| 9993 | 1.91 |
| 9999 | 2.23 |

**S11 Table. Gazelle metapodial anatomical measurements (mm)**, after von den Driesch 1976. BD—Greatest breadth of distal end; MCHDW—Width of distal condyle; MCSC—With of distal trochlea.

| **SPECIMEN #** | **BD** | **MCHDW** | **MCSC** |
| --- | --- | --- | --- |
| **8476** | 19.60 |  |  |
| **8494** | 20.93 | 9.06 | 11.50 |
| **8010** |  | 9.19 | 9.92 |
| **8051** |  | 9.02 |  |
| **8052** |  | 8.44 | 9.50 |
| **8087** |  | 9.64 | 10.57 |
| **8099** |  | 9.80 |  |
| **8100** |  | 9.52 |  |
| **8124** |  | 10.07 | 10.80 |
| **8136** |  | 9.04 | 10.86 |
| **8410** |  | 10.69 | 10.53 |
| **8411** |  | 9.15 | 11.53 |
| **8413** |  | 8.18 | 11.30 |
| **8416** |  | 9.31 | 9.94 |
| **8421** |  | 9.12 | 10.13 |
| **8422** |  | 8.96 | 11.08 |
| **8423** |  | 8.29 | 10.76 |
| **8429** |  | 10.62 | 11.73 |
| **8430** |  | 11.11 | 11.40 |
| **8432** |  | 9.58 | 11.65 |
| **8437** |  | 8.03 |  |
| **8439** |  | 8.59 |  |
| **8449** |  | 8.64 | 10.78 |
| **8450** |  | 9.75 | 11.60 |
| **8451** |  | 9.06 |  |
| **8452** |  | 9.59 | 9.84 |
| **8462** |  | 9.91 | 10.88 |
| **8466** |  | 9.54 | 11.81 |
| **8468** |  | 9.18 | 10.22 |
| **8473** |  | 8.63 |  |
| **8482** |  | 9.11 | 11.76 |
| **8483** |  | 8.37 | 10.10 |
| **8486** |  | 9.73 | 10.43 |
| **8490** |  | 8.55 | 10.71 |
| **8496** |  | 10.37 | 11.49 |
| **8501** |  | 9.12 |  |
| **8502** |  | 8.78 |  |
| **8506** |  | 10.75 | 11.81 |
| **9920** |  | 6.53 |  |
| **10051** |  | 11.49 |  |

**Bibliography:**

Amiranashvili NG. Differences in shell morphology of *Testudo graeca* and *Testudo hermanni*, based on material from Bulgaria. Amphibia-Reptilia. 2000; 21(1):67-81.

Binford LR. Nunamiut ethnoarchaeology. New York: Academic Press; 1978.

Lam YM, Chen X, Pearson OM. Intertaxonomic variability in patterns of bone density and the differential representation of bovid, cervid, and equid elements in the archaeological record. Am Antiq. 1999; 64(2): 343-62.

Lyman RL. Bone density and differential survivorship of fossil classes. J Anthropol Archaeol. 1984; 3(4): 259-99.

Lyman RL. Quantitative units and terminology in zooarchaeology. Am Antiq. 1994; 59(1): 36-71.

Munro ND, Bar-Oz G, Stutz AJ. Aging mountain gazelle (*Gazella gazella*): refining methods of tooth eruption and wear and bone fusion. J Archaeol Sci. 2009; 36(3): 752-63.

von den Driesch A. A guide to the measurement of animal bones from archaeological sites: as developed by the Institut für Palaeoanatomie, Domestikationsforschung und Geschichte der Tiermedizin of the University of Munich. Peabody Museum Press; 1976.
